# Long noncoding RNA ENST00000436340 promotes podocyte injury in diabetic kidney disease by facilitating the association of PTBP1 with RAB3B

**DOI:** 10.1038/s41419-023-05658-7

**Published:** 2023-02-15

**Authors:** Jinxiu Hu, Qimeng Wang, Xiaoting Fan, Junhui Zhen, Cheng Wang, Huimin Chen, Yingxiao Liu, Ping Zhou, Tingwei Zhang, Tongtong Huang, Rong Wang, Zhimei Lv

**Affiliations:** 1 Department of Nephrology, Shandong Provincial Hospital, Shandong University, Jinan, Shandong 250021 China; 2 Department of Pathology, School of Medicine, Shandong University, Jinan, Shandong 250021 China; 3 Department of Nephrology, Shandong Provincial Hospital Affiliated to Shandong First Medical University, Jinan, Shandong 250021 China

**Keywords:** Long non-coding RNAs, Diabetic nephropathy

## Abstract

Dysfunction of podocytes has been regarded as an important early pathologic characteristic of diabetic kidney disease (DKD), but the regulatory role of long noncoding RNAs (lncRNAs) in this process remains largely unknown. Here, we performed RNA sequencing in kidney tissues isolated from DKD patients and nondiabetic renal cancer patients undergoing surgical resection and discovered that the novel lncRNA ENST00000436340 was upregulated in DKD patients and high glucose-induced podocytes, and we showed a significant correlation between ENST00000436340 and kidney injury. Gain- and loss-of-function experiments showed that silencing ENST00000436340 alleviated high glucose-induced podocyte injury and cytoskeleton rearrangement. Mechanistically, we showed that fat mass and obesity- associate gene (FTO)-mediated m6A induced the upregulation of ENST00000436340. ENST00000436340 interacted with polypyrimidine tract binding protein 1 (PTBP1) and augmented PTBP1 binding to RAB3B mRNA, promoted RAB3B mRNA degradation, and thereby caused cytoskeleton rearrangement and inhibition of GLUT4 translocation to the plasma membrane, leading to podocyte injury and DKD progression. Together, our results suggested that upregulation of ENST00000436340 could promote podocyte injury through PTBP1-dependent RAB3B regulation, thus suggesting a novel form of lncRNA-mediated epigenetic regulation of podocytes that contributes to the pathogenesis of DKD.

## Introduction

Diabetic kidney disease (DKD), a progressive kidney disease, is a major complication associated with diabetes and has become the leading cause of chronic kidney disease in China [1, 2]. Although the disease was defined many years ago, there are no effective therapies to date. Overall, current treatments such as glucose or blood pressure control achieve only partial renoprotection [3], increasing the need for novel therapeutic approaches.

Podocytes are terminally differentiated epithelial cells that are located outside the glomerular capillaries and are an important component of the glomerular filtration barrier. Studies have confirmed that podocyte dysfunction or injury is the core event in the occurrence and progression of DKD [4]. Thus, identifying the key factors that mediate podocyte injury will provide important insights into the understanding of DKD pathogenesis.

Long noncoding RNAs (lncRNAs) are a class of RNAs longer than 200 nucleotides in length and have limited or no protein-coding potential [5]. Increasing evidence has demonstrated that lncRNAs play vital roles in kidney diseases, including DKD [6, 7]. To search for new lncRNAs involved in podocyte injury, we performed gene expression profiling in kidney tissues isolated from DKD patients and nondiabetic renal cancer patients undergoing surgical resection by RNA sequencing. We identified that ENST00000436340 was significantly upregulated in the kidney tissues of DKD patients. However, its function and detailed molecular mechanisms in DKD are completely undefined.

In the present study, we identified an ENST00000436340-PTBP1-RAB3B regulatory network involved in cytoskeleton rearrangement and GLUT4 translocation, which facilitates podocyte injury in DKD. We revealed a novel mechanism for DKD pathogenesis, which will provide insights into the prevention and treatment of DKD in the future.

## Materials and methods

### Human samples

Tissues, including kidney biopsy tissues from DKD patients and normal kidney tissues from nondiabetic renal cancer patients undergoing surgical resection, were obtained from the Department of Nephrology, Shandong Provincial Hospital, Shandong University. Serum samples obtained from DKD patients and normal controls were also obtained from the Department of Nephrology, Shandong Provincial Hospital, Shandong University.

Our study protocol was approved by the Clinical Research Ethics Committee of Shandong Provincial Hospital, Shandong University, in agreement with the guidelines set forth by the Declaration of Helsinki. All participants signed informed consent forms prior to using the tissues and serum samples for scientific research.

### RNA sequencing and data analyses

Three kidney biopsy tissues from DKD patients and 3 normal kidney tissues from nondiabetic renal cancer patients undergoing surgical resection were used for RNA sequencing. Total RNA was extracted from kidney tissues using TRIzol (Invitrogen, USA) according to the manufacturer’s protocol. RNA purity was assessed using an ND-1000 Nanodrop. Each RNA sample had an A260:A280 ratio above 1.8 and an A260:A230 ratio above 2.0. RNA integrity was evaluated using the Agilent 2200 TapeStation (Agilent Technologies, USA), and each sample had an RIN above 7.0. Ribosomal RNA was removed using EpicentreRibo-Zero rRNA Removal Kit (Illumina, USA) and fragmented to approximately 200 bp. Subsequently, the purified RNAs were subjected to first-strand and second-strand cDNA synthesis following by adaptor ligation and enrichment with a low cycle according to the instructions of the NEBNext® Ultra™ RNA Library Prep Kit for Illumina (NEB, USA). The purified library products were evaluated using the Agilent 2200 TapeStation and Qubit® 2.0 (Life Technologies, USA) and then diluted to 10 pM for cluster generation in situ on the pair-end flow cell followed by sequencing (2 × 150 bp) HiSeq3000. Raw fastq sequences were treated with Trimmomatic tools (v0.36), and sequencing read quality was inspected using FastQC software. The statistically significant DE genes were obtained by an adjusted P value threshold of <0.05 and |log_2_(fold change)| >1 using DEGseq2 software. The data of DEncRNAs and its adjacent (100KB) DEmRNAs were integrated to obtain the potential cis-regulated target genes of lncRNAs. For the prediction of trans-regulation, sequences of DEncRNAs and DEmRNAs were extracted and preliminarily screened using BLAST software (e < 1E−5). Then, RNAplex software was used to screen again to identify possible target genes of lncRNAs.

### 5′ and 3′ rapid amplification of cDNA ends (RACE) analysis

Total RNA was isolated using the TRIzol Plus RNA Purification Kit (Invitrogen). 5′ RACE and 3′ RACE analysis were performed by Shanghai DianXi Biotechnology (Shanghai, China). The following gene-specific primers (GSP) were used for PCR: 5′-GGGAGTTGATTGAGGCTGCAGTGAGCTATGG-3′ (5′ RACE GSP1), 5′-CCTGCAGCCTGATCTTCCTGGCACCGAAG-3′ (5′ RACE GSP2), 5′-CCGTGTGCTTCGGTGCCAGGAAGA-3′ (3′ RACE GSP1), 5′-GCTCACTGCAGCCTCAATCAACTCCCGGA-3′ (3′ RACE GSP2).

### Northern blot analysis

Northern blot analysis was performed to characterize the full length of ENST00000436340 and was conducted by Shanghai DianXi Biotechnology (Shanghai, China). The primers used for PCR were as follows: β-actin Forward 5′-CCCAGCCATGTACGTTGCT-3′, Reverse 5′-CTGTGTTGGCGTACAGGTCTT-3′; ENST00000436340 Forward 5′-CCAGACCGGTTAGATCACAAG-3′, Reverse 5′-ATGGTGCGGTGGCTCAGT-3′.

### Cell culture

Conditionally immortalized human podocytes (HPCs) were provided by Peter Mundel from Albert Einstein College of Medicine at Bronx, New York [8]. The podocytes were maintained in RPMI 1640 medium (Gibco, USA) supplemented with 10% FBS and 1% penicillin/streptomycin. Cells were cultured at 37 °C in a humidified incubator with 5% CO_2_. To mimic normal physiological and diabetic pathological environments, podocytes were stimulated with normal concentrations of D-glucose medium (5.5 mmol/l glucose, LG), high concentrations of D-glucose medium (30 mmol/l glucose, HG), and high osmotic medium (5.5 mmol/l glucose plus 24.5 mmol/l mannitol, HO). Insulin (Solarbio, Beijing, China) was dissolved in dilute hydrochloric acid, and the cells were treated with a final concentration of 100 nM.

### Cell transfection and infection

The overexpression plasmid targeting ENST00000436340, RAB3B, and empty vector were constructed and synthesized by Cyagen (Guangzhou, China). The lentivirus small hairpin RNA (shRNA) sequences targeting FTO were synthesized by Genomeditech (Shanghai, China). si-ENST00000436340 and negative control were purchased from RiboBio Biotechnology (Guangzhou, China). si-RAB3B siRNA, si-PTBP1 siRNA, si-GLUT4 siRNA, and negative control were purchased from Genomeditech (Shanghai, China). For transient transfection, cells were transfected with plasmids encoding target sequences or siRNAs using Lipofectamine 3000 reagent (Invitrogen, USA) according to the protocol recommended by the manufacturer. For stable infections, cells were infected with sh-FTO lentiviruses in the presence of polybrene (10 mg/ml) at a multiplicity of infection (MOI) of 50. Stable clones were selected by puromycin. The sequences used are provided in Table S1.

### RNA extraction and real-time PCR analysis

Total RNA was extracted from cultured cells and kidney tissues using TRIzol reagent (Takara, Japan). For serum RNA, RNA was extracted using a plasma/serum-free RNA extraction kit (BIOG Biotechnology, China). RNA purity was assessed using a NanoDrop-2000 spectrophotometer (Thermo Fisher Scientific, USA), and cDNA was synthesized using the PrimeScript™ RT reagent Kit with gDNA Eraser (Takara, Japan). Changes in RNA expression were quantitatively analyzed using TB Green® Premix Ex Taq™ II (TaKaRa, Japan) on a Roche LightCycler® 480II, and β-actin was used as an internal reference control. All the reactions were repeated in triplicate. The primers used for real-time PCR are shown in Table S2.

### Western blot

Total proteins were extracted with RIPA lysis buffer (Beyotime, China), quantified with a BCA Protein Assay Kit (Beyotime, China), and then subjected to sodium dodecyl sulfate-polyacrylamide gel electrophoresis (SDS-PAGE). Separated proteins were transferred onto PVDF membranes (Millipore, USA), which were incubated with 5% fat-free milk for 1 h at room temperature and incubated with corresponding primary antibodies at 4 °C overnight and subsequently incubated with horseradish peroxidase-conjugated secondary antibodies at room temperature for 1 h. Signals were detected by ECL reagent (Millipore, USA) and imaged by the Amersham Imager 600 (GE, USA). β-actin was used as an internal control for total proteins. The following antibodies were used for Western blot: anti-Desmin (Abcam, USA), anti-ZO-1 (Invitrogen, USA), anti-RAB3B (Proteintech, China), anti-PTBP1 (Cell Signaling Technology, USA).

### Immunofluorescence staining

Podocytes grown in 6-well slides were fixed with 4% paraformaldehyde and blocked with 5% BSA for 30 min at room temperature. Then, the cells were incubated with primary antibodies against synaptopodin, Desmin, and ZO-1 at 4 °C overnight. Subsequently, Alexa Fluor® 594-conjugated donkey anti-rabbit IgG (1:200, Abcam, USA) or Alexa Fluor® 488-conjugated goat anti-rabbit IgG (1:200, Abcam, USA) was applied for 1 h at 37 °C. F-actin was stained with 10 μg/ml FITC-phalloidin (Sigma, USA) according to the manufacturer’s instructions. After incubation with DAPI, the cells were observed with a fluorescence microscope (Leica, Germany).

### Transwell migration assays

RPMI 1640 medium supplemented with 10% FBS (600 μl) was seeded into the lower chamber of 24-well Transwells (Corning, USA), and 0.5 × 10^4^ podocytes exposed to different treatments were suspended in 200 μl serum-free RPMI 1640 medium and added to the upper chamber. After incubation for 48 h at 37 °C, podocytes were fixed with 4% paraformaldehyde, permeabilized with 0.1% Triton X-100, and stained with hematoxylin. Finally, the migrated cells were observed and counted in three random fields under a light microscope (Leica, Germany).

### RNA fluorescence in situ hybridization

FISH probe specific to ENST00000436340 was designed and synthesized by RiboBio Biotechnology (Guangzhou, China), and hybridization was performed using the Ribo^TM^ Fluorescent in Situ Hybridization Kit according to the protocol provided by the manufacturer. In detail, cells were fixed with 4% paraformaldehyde, permeabilized with 0.3% Triton × 100, and then cultured with a specific probe overnight. All images were captured using a fluorescence microscope (Leica, Germany). All colocalization analysis were performed using ImageJ/Fiji (N.I.H., Bethesda, MD).

### Separation of nuclear and cytoplasmic fractions

Nuclear and cytosolic fractions were separated using a PARIS kit (Am1921; Thermo Fisher Scientific, USA) according to the manufacturer’s instructions. The expression levels of ENST00000436340, GAPDH, and U6 in the cytoplasm or nucleus of podocytes were then detected using real-time PCR.

### RNA pulldown

The interaction between ENST00000436340 and PTBP1 was determined by RNA pulldown assay. Briefly, the lncRNA ENST00000436340 was transcribed in vitro with T7 RNA polymerase and biotin-labeled with a Pierce^TM^ RNA 3′ End Desthiobiotinylation Kit (Thermo Fisher Scientific, USA). Then, the cell lysate was incubated with purified biotinylated sense or antisense ENST00000436340 probe mixed with streptavidin agarose beads for 1 hour at 4 °C with rotation. After washing and eluting, a Western blot was used to detect the recruited proteins.

### RNA immunoprecipitation (RIP) assay

The RIP assay was conducted by using a Magna RIP RNA-Binding Protein Immunoprecipitation Kit (Millipore, USA) according to the manufacturer’s instructions. Briefly, approximately 1 × 10^7^ cells were washed with ice-cold PBS and then collected and lysed in complete RIP lysis buffer. Next, cell lysates were incubated with magnetic beads coated with the indicated antibodies at 4 °C for 3 h to overnight. Then, the RNA/antibody/protein complex was washed with cold RIP Wash Buffer, resuspended in proteinase K buffer, and incubated at 55 °C for 30 min to digest the protein. Then, phenol:chloroform: isoamyl alcohol was added to separate the RNA. Then, salt solution I, salt solution II, precipitate enhancer, and absolute ethanol were used to precipitate the RNA at −80 °C, followed by the analysis of immunoprecipitated RNA via real-time PCR.

### Methylated RNA immune‑precipitation (MeRIP) assay

Total RNA was isolated from podocytes, and a Magna MeRIP™ m6A Kit (Millipore, USA) was used to immunoprecipitate chemically fragmented RNA (~100 nucleotides) followed by magnetic immunoprecipitation with a monoclonal antibody against m6A. After immunoprecipitation, isolated RNA fragments can be subjected to real-time PCR.

### GLUT4 assay

The constructions of the GLUT4 carrying an HA epitope in the extracellular loop (HA-GLUT4) or EGFP-tagged-GLUT4 (GLUT4-EGFP) were generated as described previously [9, 10]. To detect cell-surface GLUT4, cells were plated in 6-well slides followed by different interventions. After 24 h of transfection, the cells were serum-starved for 2 h and then either treated or untreated with 100 nM insulin (Solarbio, China) for 30 min. After fixation with 4% paraformaldehyde, the cells were blocked with 5% BSA for 30 min at room temperature and incubated with anti-HA monoclonal antibody (1:1000, Cell Signaling Technology, USA) overnight at 4 °C, then washed three times and surface-bound monoclonal antibody detected using Alexa Fluor® 488-conjugated goat anti-rabbit IgG (1:200, Abcam, USA). Images were collected using a fluorescence microscope (Leica, Germany) and the mean fluorescence intensity was analyzed using ImageJ/Fiji software (N.I.H., Bethesda, MD). For co-localization assay, podocytes were co-transfected with exogenously expressed RAB3B-mCherry and GLUT4-EGFP. After washing with PBS and fixation with 4% paraformaldehyde, the cells were incubated with DAPI, and observed with a fluorescence microscope. Pearson’s correlation coefficient between RAB3B and GLUT4 was computed using the colocalization Finder of the ImageJ/Fiji software (N.I.H., Bethesda, MD).

### Statistical analysis

Statistical analysis was carried out using SPSS software, GraphPad Prism, ImageJ/Fiji, and Adobe Photoshop. Differences were assessed with Student’s *t*-test, one-way analysis of variance (ANOVA), or the chi-square test. Spearman correlation analysis was carried out to evaluate the correlation between serum ENST00000436340 levels and clinical parameters. *P* < 0.05 was considered significant. All experiments were performed at least in triplicate if not mentioned.

## Results

### Identification of ENST00000436340 by analyzing RNA sequencing data

**Fig. 1:**
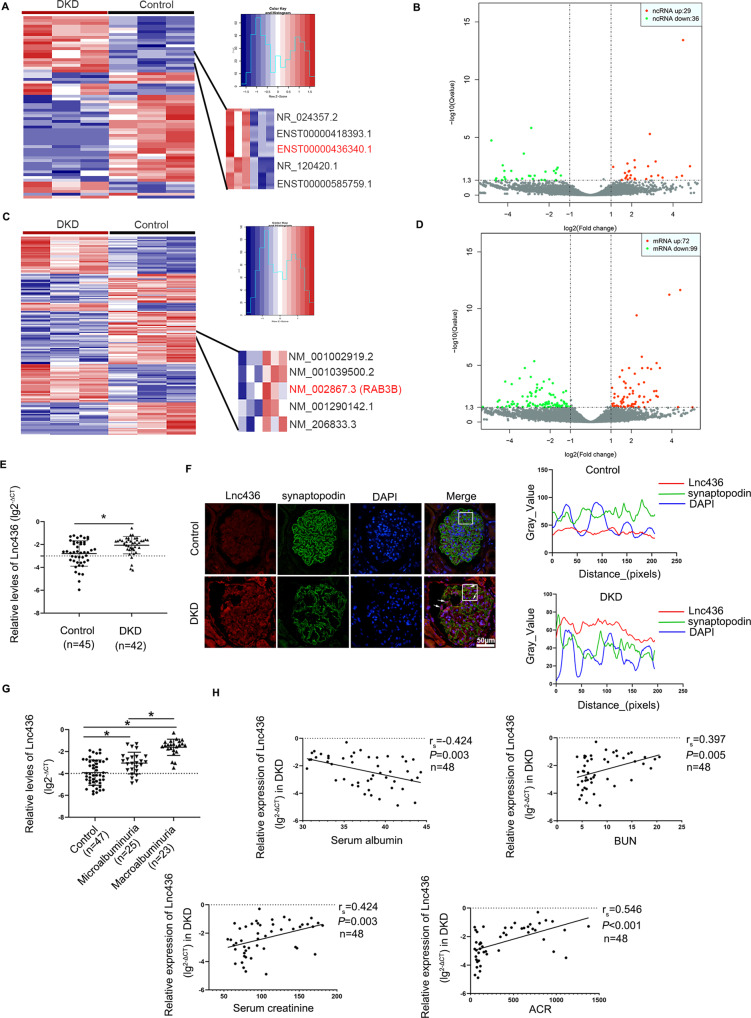
LncRNA ENST00000436340 was upregulated in human DKD. Hierarchical clustering analysis of DEncRNAs (**A**) and DEmRNAs (**C**) between DKD and control groups. The horizontal axis represents the names of the samples. The vertical axis represents the names of DEncRNAs (**A**) and DEmRNAs (**C**). Each column indicates a sample and each row indicates a gene. The color scale indicates log10 (RPKM + 1) and intensity increases from blue to red, which indicates down- and up-regulation, respectively. A volcano plot exhibiting the DEncRNAs (**B**) DEmRNAs (**D**) in which horizontal axis represents the fold changes of RNA expression in different simples. The vertical axis represents the statistical significance of the differentially expressed RNAs. Horizontal dotted line refers to Q value (the corrected *P* value) = 0.05; vertical dotted lines refer to 2-fold changes in upregulation or downregulation; red points indicate that RNAs are significantly upregulated while green points indicate significantly downregulated, and gray shows no significant differences. **E** Real-time PCR detects the expression of Lnc436 in 45 kidney biopsy tissues from DKD patients and 42 normal kidney tissues from non-diabetic renal cancer patients undergoing surgical resection. ^*^P < 0.05 vs Control. **F** Representative images of Lnc436 and podocyte-specific marker synaptopodin in the glomeruli of kidney. Arrows indicate colocalization of Lnc436 and synaptopodin. Plots of pixel intensity of white boxed region from merged images (right). Scale bar = 50 μm. **G** Real-time PCR detects the serum Lnc436 levels in Control (*n* = 47), microalbuminuria (*n* = 25), and macroalbuminuria (*n* = 23). ^*^P < 0.05 vs. Control. **H** The correlation between serum ENST00000436340 levels and serum albumin, BUN, serum creatinine, and ACR. Lnc436: LncRNA ENST00000436340.

To identify differentially expressed ncRNAs (DEncRNAs) and differentially expressed mRNAs (DEmRNAs) that are closely associated with DKD, we performed RNA sequencing using kidney tissues from renal biopsies of 3 DKD patients and normal tissues from 3 nondiabetic renal cancer patients undergoing surgical resection. The characteristics of the participants are shown in Fig. S1A and Table S3. With the filtering criteria of |log_2_FoldChange|>1 and Q-value <0.05, 65 DEncRNAs (29 upregulated and 36 downregulated in DKD) and 171 DEmRNAs (72 upregulated and 99 downregulated in DKD) were identified (Fig. 1A–D). Through KEGG enrichment analyses, we found that differentially expressed genes were significantly related to the calcium signaling pathway, cAMP signaling pathway, MAPK signaling pathway, metabolic pathways, etc. (Fig. S1B), indicating that these pathways may play important roles in the progression of DKD.

Considering that the lncRNA ENST00000436340 was significantly upregulated in DKD compared with the controls, we focused on ENST00000436340 for further study to explore its role in DKD progression. According to *Ensembl*, ENST00000436340 mapped to chromosome 10 with 3 exons and was 1309 bp in length (Fig. S1C). The coding potential calculator (CPC2) [11] and coding-potential assessment tool (CPAT) [12] confirm that ENST00000436340 is a noncoding RNA due to its negligible protein-coding potential (Fig. S1D).

Intriguingly, an evolutionary analysis of the ENST00000436340 nucleotide sequence using the UCSC database (https://genome.ucsc.edu/) showed low conservation in mouse, but high conservation in chimpanzees, gorillas, and Rhesus, which are phylogenetically most closely related to humans (Fig. S1E), suggesting that ENST00000436340 might be a primate-specific lncRNA.

### ENST00000436340 is upregulated in human DKD

To validate the reliability of the expression level of ENST00000436340 obtained from RNA sequencing, we carried out real-time PCR in kidney tissues of DKD patients and confirmed the expression of ENST00000436340 (Fig. 1E). RNA-Fluorescence in Situ Hybridization (RNA-FISH) showed that the expression of ENST00000436340 was significantly increased in the glomeruli of patients with DKD. In addition, costaining with synaptopodin, a marker protein for podocytes, showed that ENST00000436340 colocalizes with podocytes, and the expression of ENST00000436340 in podocytes was upregulated when podocytes were injured (Fig. 1F), indicating that the overexpression of ENST00000436340 may be related to podocyte injury. However, we did not observe a significant difference in ENST00000436340 in podocytes from other glomerular diseases, such as minimal change disease (MCD) and membranous nephropathy (MN) (Fig. S2).

Currently, studies have shown that circulating lncRNAs are stably present in plasma, serum, and urine and could serve as noninvasive biomarkers for predicting the diagnosis and prognosis of diseases or targets for drug treatment [13–16]. Therefore, in our study, we examined its expression in 95 serum samples, including 47 control (urine ACR < 30 mg/g), 25 microalbuminuria (30 mg/g<ACR < 300 mg/g), and 23 macroalbuminuria (ACR > 300 mg/g) samples. The basic information and related biochemical indicators are shown in Table S4. There were no significant differences in age, gender, TC, or LDL among the groups, but the BP, HbAc1, BUN, serum creatinine, TG, and ACR of patients with DKD were significantly higher than those of controls (*P* < 0.05). In addition, compared with the control, the serum albumin, HDL, and eGFR of DKD were significantly reduced (*P* < 0.05). The results of real-time PCR revealed the aberrant overexpression of ENST00000436340 in the serum of patients with DKD compared with the control group. In addition, we observed that the expression level of ENST00000436340 in patients with macroalbuminuria was higher than the expression level of ENST00000436340 in patients with microalbuminuria (Fig. 1G), suggesting that the expression of ENST00000436340 might be related to the severity of urine albumin levels. Additionally, we evaluated the correlation between serum ENST00000436340 levels and clinical parameters of kidney function (Table S5). Spearman correlation analysis showed that serum albumin (r_s_ = −0.424, *P* = 0.003), BUN (r_s_ = 0.397, *P* = 0.005), serum creatinine (r_s_ = 0.424, *P* = 0.003), and ACR (r_s_= 0.546, *P* < 0.001) were correlated with serum ENST00000436340, and the correlation with ACR was the most significant (Fig. 1H). To further verify the specificity of the expression changes of ENST00000436340 in DKD, we also detected its expression level in the serum of CKD patients, and the results showed no statistical significance between CKD patients and controls. Taken together, lncRNA ENST00000436340 increases are specific to DKD and might play an indispensable role in kidney injury.

### ENST00000436340 was involved in high glucose-induced podocyte injury and cytoskeleton rearrangement

**Fig. 2:**
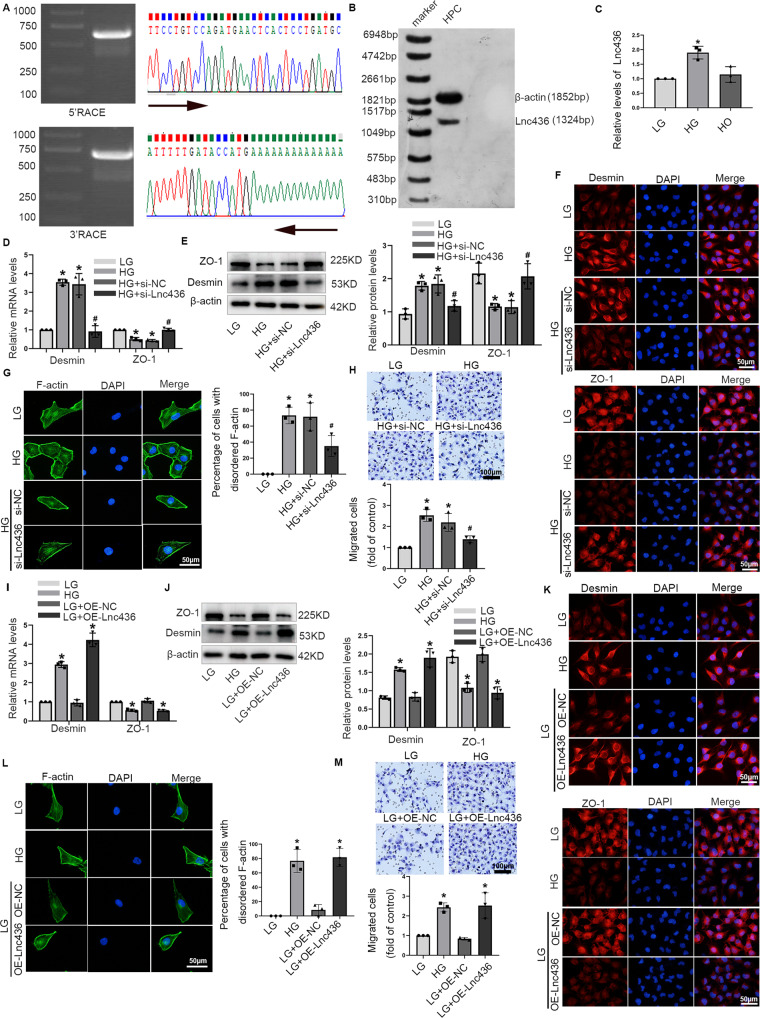
ENST00000436340 was involved in high glucose-induced podocyte injury and cytoskeleton rearrangement. **A** 5′ and 3′rapid amplification of cDNA ends (RACE) assays in podocytes to detect the whole sequence of ENST00000436340. Left: a gel electrophoresis image of PCR products from the 5′-RACE and 3′-RACE assays. Right: sequencing of PCR products. **B** Northern blot analysis to confirm the length and expression of the ENST00000436340 in podocytes. β-actin was used as a loading control. **C** Real-time PCR analyses show the level of ENST00000436340 in different groups of podocytes. **D** Real-time PCR analyses show the mRNA levels of Demin and ZO-1 in podocytes treated with ENST00000436340 siRNA (si-Lnc436) or negative control (si-NC) in the presence of HG. **E** Western blot analyses show the protein levels of Desmin and ZO-1 in podocytes treated with ENST00000436340 siRNA (si-Lnc436) or negative control (si-NC) in the presence of HG. **F** Representative immunofluorescence images of Desmin, and ZO-1 in podocytes treated with ENST00000436340 siRNA (si-Lnc436) or negative control (si-NC) in the presence of HG. Scale bar = 50 μm. **G** Representative immunofluorescence images of F-actin in podocytes treated with ENST00000436340 siRNA (si-Lnc436) or negative control (si-NC) in the presence of HG. Scale bar = 50 μm. **H** Representative migration results of podocytes treated with ENST00000436340 siRNA (si-Lnc436) or negative control (si-NC) in the presence of HG. Scale bar = 100 μm. **I** real-time PCR analyses show the mRNA level of Demin and ZO-1 in podocytes treated with ENST00000436340 overexpression plasmid (OE-Lnc436) or control (OE-NC) vectors in the presence of LG. **J** Western blot analyses show the protein level of Desmin and ZO-1 in podocytes treated with ENST00000436340 overexpression plasmid (OE-Lnc436) or control (OE-NC) vectors in the presence of LG. **K** Representative immunofluorescence images of Desmin, and ZO-1 in podocytes treated with ENST00000436340 overexpression plasmid (OE-Lnc436) or control (OE-NC) vectors in the presence of LG. Scale bar = 50 μm. **L** Representative immunofluorescence images of F-actin in podocytes treated with ENST00000436340 overexpression plasmid (OE-Lnc436) or control (OE-NC) vectors in the presence of LG. Scale bar = 50 μm. **M** Representative migration results of podocytes treated with ENST00000436340 overexpression plasmid (OE-Lnc436) or control (OE-NC) vectors in the presence of LG. Scale bar = 100 μm. Data are shown as mean ± SD. *P < 0.05 vs LG, ^#^*P* < 0.05 vs. HG. The experiment was performed in triplicate.

To clarify whether ENST00000436340 was involved in podocyte injury, we first characterized ENST00000436340 by rapid amplification of the 5′ and 3′ cDNA ends (RACE) assays (Fig. 2A) and Northern blot (Fig. 2B). The results validated that full-length ENST00000436340 RNA was 1324 bp in length. Subsequently, we assessed the level of ENST00000436340 in high glucose-treated podocytes using real-time PCR, and the results demonstrated that high glucose treatment resulted in an increase in ENST00000436340 expression (Fig. 2C). Next, we carried out real-time PCR, Western blot and immunofluorescence staining to further verify the effects of high glucose on podocytes, and we observed that high glucose upregulated the expression of the podocyte injury marker Desmin and downregulated the expression of ZO-1 (Fig. S3A–C). In addition, FITC-phalloidin staining demonstrated that the F-actin cytoskeleton, which appears as bundles along the cell axis under low glucose conditions, was disorganized, collapsed and peripherally located in high glucose-treated cells (Fig. S3D). In addition, the results of the Transwell assay revealed that the migration ability of podocytes was significantly enhanced when treated with high glucose (Fig. S3E).

To further investigate the role of ENST00000436340 in high glucose-induced podocyte injury, podocytes were transfected with ENST00000436340 siRNA or ENST00000436340 overexpression plasmid (Fig. S3F) to downregulate or upregulate the expression of ENST00000436340, and the results demonstrated that silencing of ENST00000436340 reversed the upregulation of Desmin, downregulation of ZO-1, cytoskeleton rearrangement, and enhanced the migration ability induced by high glucose, alleviating podocyte injury (Fig. 2D–H), while overexpression of ENST00000436340 promoted the occurrence of podocyte injury and cytoskeleton rearrangement (Fig. 2I–M).

### FTO is associated with ENST00000436340 upregulation in high glucose-induced podocytes

Next, we investigated the underlying mechanism of ENST00000436340 upregulation in high glucose-induced podocytes. Considering that m6A modification modulates the expression and functions of RNAs, including lncRNAs, we detected the m6A abundance of ENST00000436340 by performing m6A RNA immunoprecipitation (MeRIP), and the results showed that m6A was highly enriched within ENST00000436340 in podocytes (Fig. S4A). FTO is a main m6A demethylase and has been reported to be involved in diabetes and kidney diseases. Real-time PCR and Western blot results showed that FTO was overexpressed in high glucose-induced podocytes (Fig. S4B, C). To explore the effects of FTO on ENST00000436340 upregulation, we knocked down the expression of FTO using lentivirus in high glucose-induced podocytes, and the knockdown efficiency is shown in Fig. S4D, E. Compared to the control, the expression of ENST00000436340 decreased with knockdown of FTO (Fig. S4B), and the m6A abundance of ENST00000436340 was upregulated upon FTO knockdown (Fig. S4A), suggesting that FTO-mediated m6A demethylation might be responsible for the upregulation of ENST00000436340, and ENST00000436340 might be a potential target of FTO. However, further study is needed to elucidate a detailed regulatory mechanism.

### ENST00000436340 participated in high glucose-induced podocyte injury by downregulating RAB3B

**Fig. 3:**
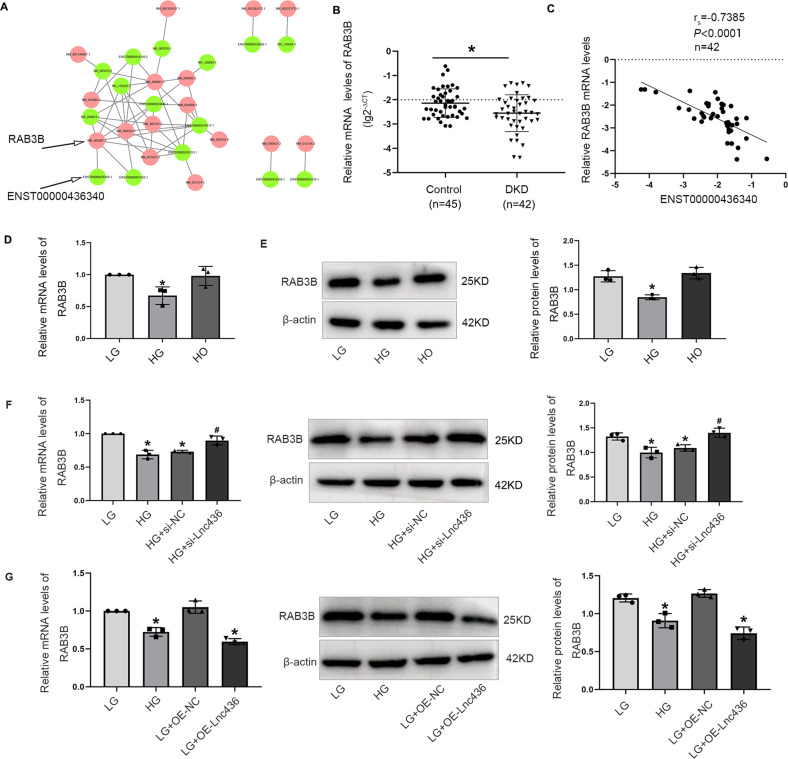
ENST00000436340 negatively regulated the expression of RAB3B. **A** DElncRNA-DEmRNA networks. The green dots represent the DElncRNAs while the red dots represent the predicted target DEmRNAs of DElncRNAs, and the lines represent the relationship between DElncRNAs and DEmRNAs. **B** Real-time PCR analyses of RAB3B mRNA expression in DKD kidney tissues. *P < 0.05 vs Control. **C** The correlation between ENST00000436340 level with RAB3B expression in kidney tissue samples. **D** Real-time PCR analyses show the mRNA levels of RAB3B in different groups of podocytes. *P < 0.05 vs. LG. **E** Western blot analyses show the protein levels of RAB3B in different groups of podocytes. *P < 0.05 vs. LG. **F** Real-time PCR and Western blot analyses show the mRNA and protein levels of RAB3B in podocytes treated with ENST00000436340 siRNA (si-Lnc436) or negative control (si-NC) in the presence of HG. *P < 0.05 vs. LG, ^#^*P* < 0.05 vs HG. **G** Real-time PCR and Western blot analyses show the mRNA and protein levels of RAB3B in podocytes treated with ENST00000436340 overexpression plasmid (OE-Lnc436) or control (OE-NC) vectors in the presence of LG. *P < 0.05 vs LG. Data are shown as mean ± SD. The experiment was performed in triplicate.

LncRNAs could play their role by regulating target mRNA expression by cis or trans, so the relationship between DElncRNAs and DEmRNAs can be established. These findings will be helpful for further research on the function of lncRNAs. In total, 5 pairs of lncRNA-mRNAs for cis-regulation and 45 pairs of lncRNA-mRNAs for trans-regulation were predicted (Table S6). Based on these results, we constructed DElncRNA-DEmRNA networks, as shown in Fig. 3A. We identified RAB3B, one of the DEmRNAs between the control and DKD groups in our RNA sequencing results (Fig. 1C), as a possible target gene of ENST00000436340, and ENST00000436340 might regulate RAB3B expression by trans. Subsequently, we verified the expression of RAB3B by performing real-time PCR and found that RAB3B was downregulated in the kidney tissues of patients with DKD (Fig. 3B), consistent with the results of RNA sequencing. Furthermore, we observed that ENST00000436340 was negatively associated with RAB3B expression (Fig. 3C). In addition, real-time PCR and Western blot results showed that RAB3B was downregulated in high glucose-treated podocytes compared to control podocytes at both the mRNA and protein levels (Fig. 3D, E). In addition, silencing ENST00000436340 upregulated the expression of RAB3B, while overexpression of ENST00000436340 downregulated the expression of RAB3B (Fig. 3F, G).

**Fig. 4:**
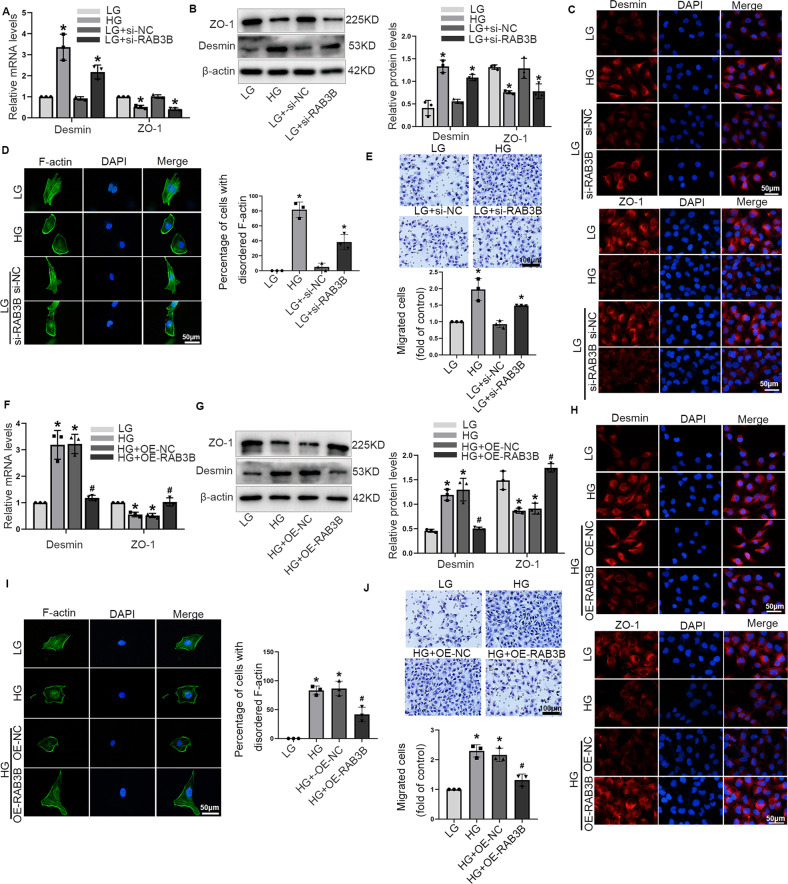
RAB3B was involved in high glucose-induced podocytes injury and cytoskeleton rearrangement. **A** Real-time PCR analyses show the mRNA levels of Demin and ZO-1 in podocytes treated with RAB3B siRNA (si-RAB3B) or negative control (si-NC) in the presence of LG. **B** Western blot analyses show the protein levels of Desmin and ZO-1 in podocytes treated with RAB3B siRNA (si-RAB3B) or negative control (si-NC) in the presence of LG. **C** Representative immunofluorescence images of Desmin and ZO-1 in podocytes treated with RAB3B siRNA (si-RAB3B) or negative control (si-NC) in the presence of LG. Scale bar = 50 μm. **D** Representative images of F-actin in podocytes treated with RAB3B siRNA (si-RAB3B) or negative control (si-NC) in the presence of LG. Scale bar = 50 μm. **E** Representative migration results of podocytes treated with RAB3B siRNA (si-RAB3B) or negative control (si-NC) in the presence of LG. Scale bar = 100 μm. **F** Real-time PCR analyses show the mRNA levels of Demin and ZO-1 in podocytes treated with RAB3B overexpression plasmid (OE-RAB3B) or control (OE-NC) vectors in the presence of HG. **G** Western blot analyses show the protein levels of Desmin and ZO-1 in podocytes treated with RAB3B overexpression plasmid (OE-RAB3B) or control (OE-NC) vectors in the presence of HG. **H** Representative immunofluorescence images of Desmin and ZO-1 in podocytes treated with RAB3B overexpression plasmid (OE-RAB3B) or control (OE-NC) vectors in the presence of HG. Scale bar = 50 μm. **I** Representative images of F-actin in podocytes treated with RAB3B overexpression plasmid (OE-RAB3B) or control (OE-NC) vectors in the presence of HG. Scale bar = 50 μm. **J** Representative migration results of podocytes treated with RAB3B overexpression plasmid (OE-RAB3B) or control (OE-NC) vectors in the presence of HG. Scale bar = 100 μm. Data are shown as mean ± SD. *P < 0.05 vs. LG, ^#^*P* < 0.05 vs. HG. The experiment was performed in triplicate.

**Fig. 5:**
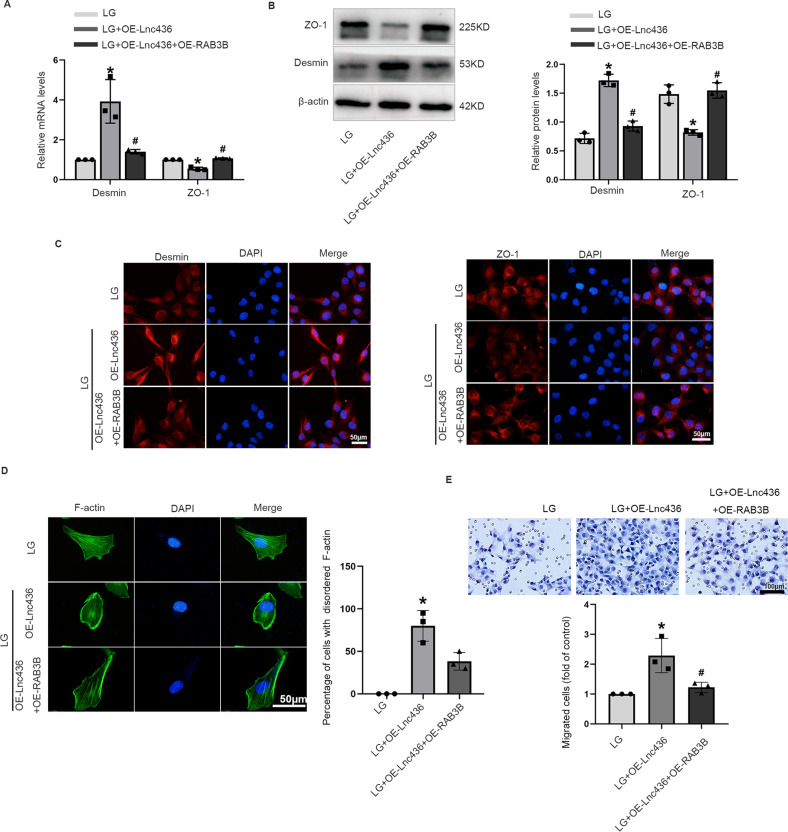
RAB3B is essential for ENST00000436340-regulated high glucose-induced podocyte injury. **A** real-time PCR analyses show the mRNA levels of Demin and ZO-1 in different groups of podocytes. **B** Western blot analyses show the protein levels of Desmin and ZO-1 in different groups of podocytes. **C** Representative immunofluorescence images of Desmin and ZO-1 in different groups of podocytes. Scale bar = 50 μm. **D** Representative images of F-actin in different groups of podocytes. Scale bar = 50 μm. (**E**) Representative migration results in different groups of podocytes. Scale bar = 100 μm. Data are shown as mean ± SD. *P < 0.05 vs. LG, ^#^*P* < 0.05 vs. LG + OE-Lnc436. The experiment was performed in triplicate.

Next, we sought to determine whether RAB3B was essential for ENST00000436340-regulated high glucose-induced podocyte injury. By performing a series of gain- and loss-of-function experiments, we found that inhibition of RAB3B upregulated the expression of Desmin, downregulated the expression of ZO-1, and promoted cytoskeleton rearrangement and migration, aggravating podocyte injury (Fig. 4A–E and Fig. S5A, B), whereas overexpression of RAB3B attenuated the above effects (Fig. 4F–J and Fig. S5C, D). Moreover, enhanced RAB3B abrogated the podocyte injury and cytoskeleton rearrangement induced by ENST00000436340 (Fig. 5).

### ENST00000436340 interacting with PTBP1

**Fig. 6:**
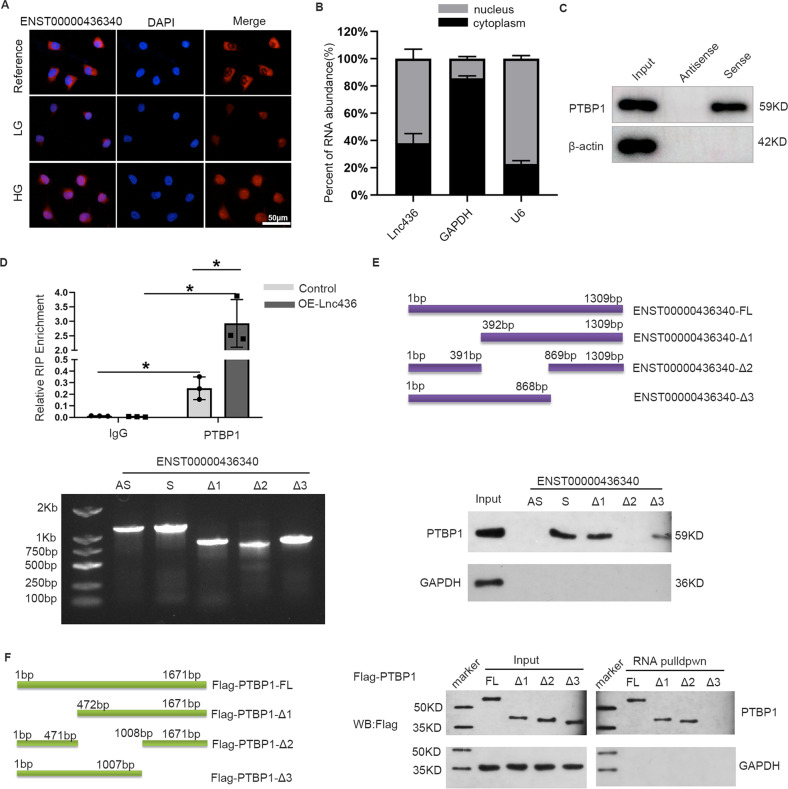
ENST00000436340 interacting with PTBP1. **A** RNA-FISH showed that ENST00000436340 was distributed both in the cytoplasm and nucleus of podocytes. 18 s was used as the reference. Scale bar = 50 μm. **B** Subcellular fractionation assays verified that ENST00000436340(Lnc436) distributed both in the cytoplasm and nucleus of podocytes. **C** RNA pulldown assay showed that PTBP1 protein was enriched by ENST00000436340 in podocytes. β-actin was used as a negative control. **D** RIP was performed using anti-PTBP1 and control IgG antibodies, followed by real-time PCR to examine the enrichment of ENST00000436340(Lnc436). **E** Serial deletions of ENST00000436340 were used in RNA pulldown assays to map the PTBP1 binding region of ENST00000436340. **F** Serial deletions of PTBP1 were used in RNA pulldown assays to determine the region in PTBP1 that binds to ENST00000436340. Data are shown as mean ± SD. The experiment was performed in triplicate.

Our results showed that ENST00000436340 exerted its role by regulating RAB3B. However, the detailed molecular mechanism had not yet been investigated. To clarify the molecular mechanism, we first evaluated the subcellular localization of ENST00000436340 in podocytes, and the results of RNA-FISH (Fig. 6A) and subcellular fractionation assays (Fig. 6B) showed that ENST00000436340 was localized mainly in the nuclei of podocytes, indicating that ENST00000436340 may function by interacting with RNA-binding proteins (RBPs). By searching the RBPDB (http://rbpdb.ccbr.utoronto.ca/index.php) and Pfam databases (http://pfam.xfam.org/), we found that PTBP1 potentially binds to both ENST00000436340 and RAB3B. Then, we conducted RNA pulldown assays followed by Western blot with PTBP1 antibody, and we found that PTBP1 bound to biotin-labeled sense-ENST00000436340 but not to antisense-ENST00000436340 (Fig. 6C). To further verify the interaction, we performed RNA immunoprecipitation (RIP) assays in podocytes transfected with ENST00000436340 overexpression or control vectors, and significant enrichment was observed in the overexpression group compared with the control group (Fig. 6D), consistent with the results of RNA pulldown.

To map the PTBP1 binding region, we divided full-length ENST00000436340 into three fragments and performed RNA pulldown assays. The results showed that the full-length, Δ1 and Δ3 fragments of ENST00000436340 directly bound to PTBP1, indicating that PTBP1 is a binding partner for the 392-868 bp region of ENST00000436340 (Fig. 6E).

To determine the region in PTBP1 that binds to ENST00000436340, we further divided full-length PTBP1 into three fragments, termed Flag-PTBP1-Δ1, Flag-PTBP1-Δ2, and Flag-PTBP1-Δ3. RNA pulldown confirmed that both the FL, Δ1, and Δ2 fragments of PTBP1 could bind to ENST00000436340, showing that residues 1008-1671 of PTBP1 were responsible for binding to ENST00000436340 (Fig. 6F).

### ENST00000436340 enhances PTBP1 binding to RAB3B mRNA to promote its degradation

PTBP1 has been reported to be able to regulate its target mRNA metabolism by controlling its stability and translation [17–19]. Based on the fact that ENST00000436340 is able to bind PTBP1, we hypothesize that the interaction between ENST00000436340 and PTBP1 may influence the effect of PTBP1 on RAB3B.

**Fig. 7:**
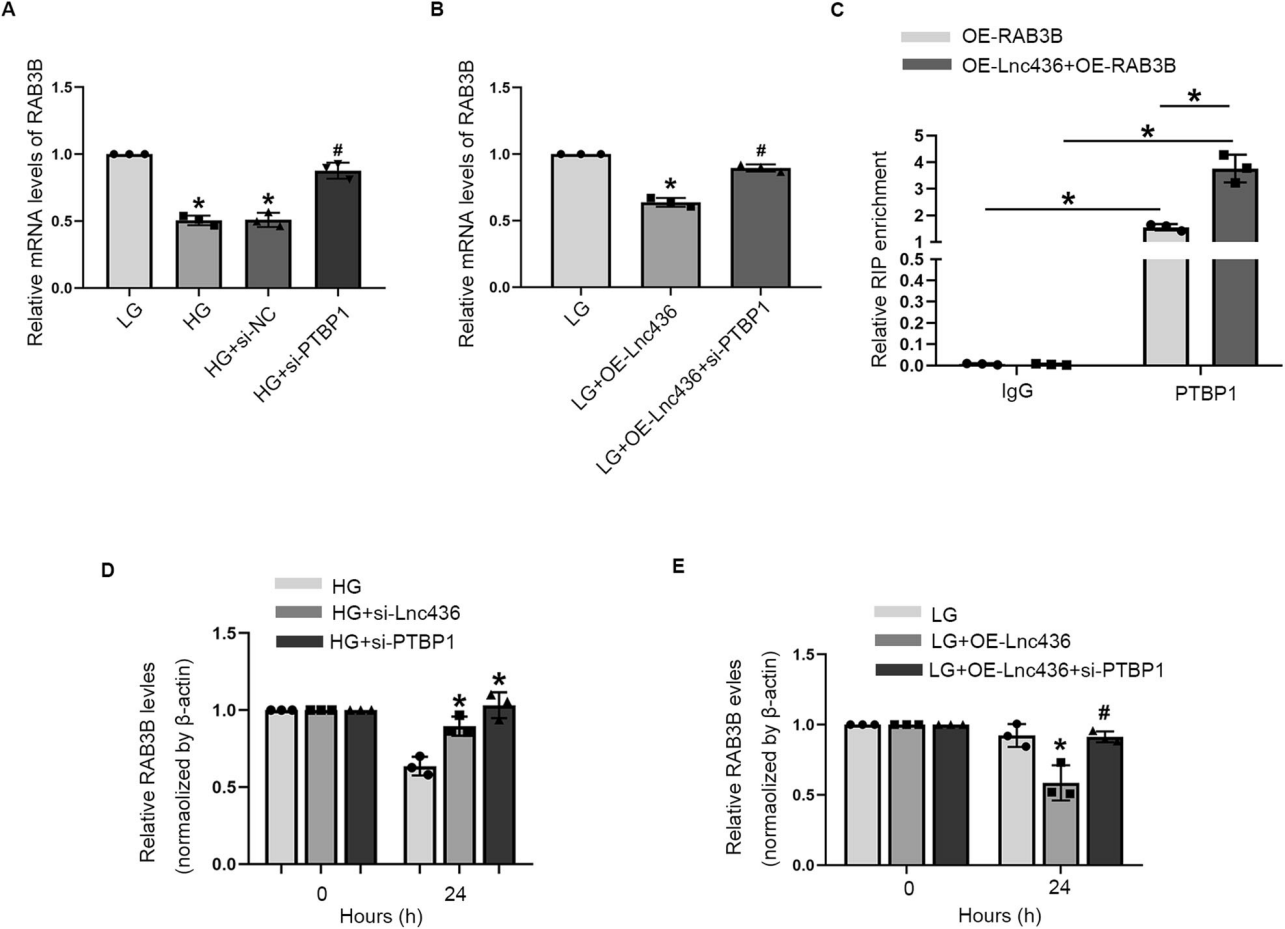
ENST00000436340 enhances PTBP1 binding to RAB3B mRNA to promote its degradation. **A** real-time PCR analyses show the mRNA levels of RAB3B in podocytes treated with PTBP1 siRNA (si-PTBP1) or negative control (si-NC) in the presence of HG. *P < 0.05 vs. LG, ^#^*P* < 0.05 vs. HG. **B** real-time PCR analyses show the mRNA levels of RAB3B in different groups of podocytes. ^*^P < 0.05 vs. LG, ^#^*P* < 0.05 vs. LG + OE-Lnc436. **C** RIP was performed to determine the interaction between RAB3B mRNA and PTBP1 protein using anti-PTBP1 or IgG in the presence or absence of ENST00000436340(Lnc436) coexpression. **D** Podocytes transfected with ENST00000436340 siRNA(si-Lnc436) or PTBP1 siRNA(si-PTBP1) were treated with ActD (5 μg/ml) for 0 h or 24 h and RAB3B mRNA abundance, relative to β-actin, was quantified by real-time PCR. *P < 0.05 vs. HG. (**E**) Podocytes transfected with ENST00000436340 overexpression plasmid vector (OE-Lnc436) alone or together with PTBP1 siRNA(si-PTBP1) were treated with ActD (5 μg/ml) for 0 h or 24 h and RAB3B mRNA abundance, relative to β-actin, was quantified by real-time PCR. *P < 0.05 vs LG, ^#^*P* < 0.05 vs LG + OE-Lnc436. Data are shown as mean ± SD. The experiment was performed in triplicate.

Real-time PCR showed that the silencing of PTBP1 upregulated RAB3B mRNA levels (Fig. 7A and Fig. S6). Moreover, the silencing of PTBP1 abrogated the ENST00000436340-induced decrease in RAB3B mRNA (Fig. 7B). Next, we performed a RIP assay in podocytes transfected with RAB3B overexpression vector in the presence or absence of ENST00000436340 coexpression, and we observed that RAB3B mRNA was enriched by PTBP1 antibody when compared with control IgG. More importantly, overexpression of ENST00000436340 significantly increased the binding of PTBP1 and RAB3B mRNA (Fig. 7C). These data collectively revealed that the binding of ENST00000436340 and PTBP1 enhanced the interaction between PTBP1 and RAB3B mRNA.

To evaluate the effects of PTBP1 on RAB3B mRNA stability, we treated HG-induced podocytes with actinomycin D (ActD, 5 μg/ml), which is widely used for the experimental blockage of the transcriptional process, and we found that silencing ENST00000436340 or PTBP1 caused decreased degradation of RAB3B mRNA (Fig. 7D). In addition, overexpression of ENST00000436340 promoted its degradation, while knockdown of PTBP1 increased its stability back to normal levels (Fig. 7E). Thus, our results proved that ENST00000436340-bound PTBP1 decreased the expression levels of RAB3B by promoting its degradation.

### GLUT4 plays a vital role in ENST00000436340/RAB3B-induced podocyte injury in DKD

**Fig. 8:**
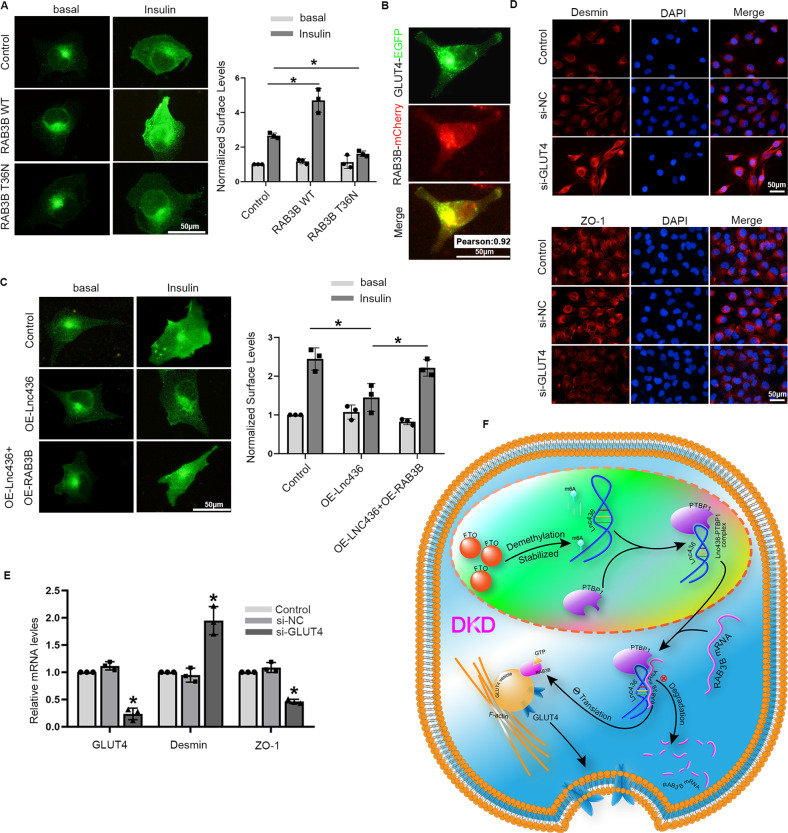
GLUT4 plays a vital role in ENST00000436340/RAB3B-induced podocyte injury in DKD. **A** Cells were transfected with HA-GLUT4 alone (Control), HA-GLUT4 and wild-type RAB3B (RAB3B WT), or HA-GLUT4 and RAB3B T36N mutant (RAB3B T36N), the surface-localized GLUT4 were detected by anti-HA antibody, and the mean fluorescence intensity was normalized to that of basal Control cells by using ImageJ/Fiji. Scale bar = 50 μm, *P < 0.05. **B** Representative images of the colocalization of RAB3B and GLUT4. Scale bar = 50 μm. Pearson’s correlation coefficient between RAB3B and GLUT4 was computed using the colocalization Finder of the ImageJ/Fiji software as indicated. **C** Cells were transfected with HA-GLUT4 alone (Control), HA-GLUT4, and ENST00000436340 overexpression plasmid without (OE-Lnc436) or with RAB3B overexpression plasmid (OE-Lnc436 + OE-RAB3B), the surface-localized GLUT4 were detected by anti-HA antibody, and the mean fluorescence intensity was normalized to that of basal Control cells by using ImageJ/Fiji. Scale bar = 50 μm, *P < 0.05. **D** Representative immunofluorescence images of Desmin and ZO-1 in different groups of podocytes. Scale bar = 50 μm. **E** real-time PCR analyses show the mRNA levels of Desmin and ZO-1 in podocytes treated with GLUT4 siRNA (si-GLUT4) or negative control (si-NC). *P < 0.05 vs Control. Data are shown as mean ± SD. The experiment was performed in triplicate. **F** Schematic model of the role of lncRNA ENST00000436340. FTO-mediated m6A induced the upregulation of ENST00000436340. ENST00000436340 interacted with PTBP1 and augmented PTBP1 binding to RAB3B mRNA and thereby promoted RAB3B mRNA degradation, caused cytoskeleton rearrangement and inhibition of GLUT4 translocation to the plasma membrane, leading to podocyte injury and DKD progression.

Considering that insulin resistance is one of the key mechanisms associated with the progression of DKD and that Rab GTPases are involved in GLUT4 trafficking [20, 21], which plays an important role in insulin resistance [22], we further discuss the role of RAB3B in insulin-stimulated GLUT4 translocation in podocytes. First, we transfected HA-GLUT4 into podocytes and then stimulated them with or without insulin. Subsequently, we used an anti-HA antibody to detect surface-localized GLUT4 via immunofluorescence staining, and we observed that surface-localized GLUT4 was significantly increased after insulin stimulation when compared with cells in the basal state, indicating that podocytes were insulin responsive and that insulin enhanced the translocation of GLUT4 to the cell surface (Fig. 8A).

Our RAB3B antibody does not work in immunofluorescence, and we were, therefore, unable to localize endogenous RAB3B in podocytes by immunofluorescence. Therefore, we cotransfected cells with exogenously expressed RAB3B-mCherry and GLUT4-EGFP, and we observed colocalization between the two proteins in the perinuclear region of podocytes (Fig. 8B). We next investigated whether RAB3B constructs affected insulin-stimulated translocation of GLUT4 to the cell surface. Wild-type and dominant-negative mutant (T36N) RAB3B-mCherry were expressed in podocytes together with HA-GLUT4. Expression of wild-type RAB3B increased insulin-stimulated translocation of GLUT4 to the plasma membrane, while expression of RAB3B T36N blunted the insulin-stimulated translocation of GLUT4 to the plasma membrane. However, the expression of wild-type RAB3B or RAB3B T36N did not have an effect on the GLUT4 distribution in the basal state (Fig. 8A).

To further confirm the role of RAB3B in insulin-stimulated GLUT4 translocation, we transfected ENST00000436340 overexpression plasmid with or without RAB3B coexpression, and we observed that enhanced RAB3B reversed the inhibition of insulin-stimulated GLUT4 translocation induced by ENST00000436340 overexpression (Fig. 8C). In addition, we observed that silencing GLUT4 promoted the occurrence of podocyte injury by performing real-time PCR and immunofluorescence staining (Fig. 8D, E). Taken together, these results demonstrate that GLUT4 plays a vital role in ENST00000436340/RAB3B-induced podocyte injury in DKD and that the GTPase activity of RAB3B is important for this process.

## Discussion

In recent years, with the rapid development of microarray chips and high-throughput sequencing technologies, the biological characteristics and functions of lncRNAs have gradually been recognized, and increasing evidence shows that abnormal expression of lncRNAs is closely related to DKD [6, 7, 13]. By performing RNA sequencing, we identified a novel lncRNA, ENST00000436340, that was upregulated in kidney tissues and serum of DKD patients. Moreover, the level of ENST00000436340 was much higher in DKD patients with macroalbuminuria than in those with microalbuminuria, and there was a positive correlation between ENST00000436340 levels and kidney injury. These findings suggest that ENST00000436340 might be a risk gene associated with the progression of DKD and can be a useful biomarker for therapy in the clinic. Acting as highly differentiated epithelial cells, podocytes are an important component of the glomerular filtration barrier, and studies have confirmed that their depletion or dysfunction is the core event for the occurrence and progression of DKD [23, 24]. In our study, we observed colocalization between ENST00000436340 and synaptopodin, an important marker protein of podocytes, by performing RNA-FISH, suggesting that ENST00000436340 may be related to podocyte injury. Gain- and loss-of-function assays showed that the knockdown of ENST00000436340 reversed high glucose-induced podocyte injury and cytoskeleton rearrangement, while overexpression of ENST00000436340 promoted the occurrence of podocyte injury, which confirmed our speculation.

Recent years have witnessed remarkable advancements of N6-methyladenosine (m6A) modification in regulating lncRNAs, such as RP11 [25], LINC00958 [26], XIST [27], and MALAT1 [28]. m6A, defined as the RNA methylation on the sixth N atom of adenylate, is the most predominant modification of mRNA in eukaryotic cells and has become a hot topic of the attention of researchers [29]. As an epigenetic modification found not only in mRNAs but also in noncoding RNAs [30–32], perturbations of m6A have been proven to play a vital role in multiple diseases, such as cancer [33], infertility [34], virus infection [35], and metabolic diseases [36]. However, the regulation of lncRNAs by m6A modification in DKD is still poorly understood. In our study, the MeRIP assay revealed that the m6A-modified ENST00000436340 transcript is present in podocytes.

Fat mass and obesity-associated protein FTO, also termed ALKBH9, is the first RNA demethylase [33] and the first GWAS‑identified obesity gene [37]. Dysregulation of FTO has been reported in various kidney diseases, such as ccRCC [38], AKI [39], urethral obstruction, and renal fibrosis [40]. Moreover, previous population-based case-control studies have suggested that FTO rs17817449 variants are associated with increased risks of chronic kidney diseases (CKD) and onset of ESRD [41], while a genome-wide association study (GWAS) meta-analysis revealed that the FTO rs56094641 locus had a significant association with susceptibility to diabetic nephropathy [42]. In this study, we observed a high enrichment of m6A within ENST00000436340 in podocytes and an increase in FTO expression in high glucose-induced podocytes, consistent with previous studies [36, 43, 44]. Moreover, the m6A demethylase FTO regulated ENST00000436340 m6A levels and its RNA expression, indicating that FTO-mediated m6A demethylation is associated with the upregulation of ENST00000436340 and that ENST00000436340 is a potential target of FTO. To our knowledge, this is the first report that the stability of lncRNA ENST00000436340 in podocytes can be regulated by m6A, expanding our understanding of lncRNAs participating in the pathogenesis of DKD. Although we preliminarily proved the role of FTO in the upregulation of ENST00000436340, the precise regulatory mechanism requires further study to be fully elucidated. The possible mechanisms involved in the FTO-regulated upregulation of ENST00000436340 may be as follows: [1] FTO affects the activity of the ENST00000436340 promoter and actives ENST00000436340 transcription; [2] FTO affects the half-life of ENST00000436340 and then increases the stability of its transcript; [3] the subcellular fractionation of ENST00000436340 is altered, which increases the nuclear accumulation and stability of nascent ENST00000436340; and [4] the expression of FTO affects the interaction between ENST00000436340 and the m6A reader protein, which subsequently inhibits its degradation.

lncRNAs can regulate target genes either in cis or in trans. In cis, lncRNAs regulate the expression of genes positioned in the vicinity of their transcription sites on the same chromosome. In trans, lncRNAs regulate the gene located on other chromosomes [45–47]. To investigate the target gene of ENST00000436340, DElncRNAs, and DEmRNAs were screened to identify possible target genes of ENST00000436340, and finally, we identified RAB3B, one of the DEmRNAs between the control and DKD groups in our RNA-sequencing results, is the possible target of ENST00000436340 and ENST00000436340 might regulate RAB3B expression by trans. Further functional experiments proved that ENST00000436340 promoted DKD progression by downregulating RAB3B.

Given the nuclear distribution of ENST00000436340 in podocytes, we further explored whether ENST00000436340 regulated RAB3B by interacting with RBPs. By searching the RBPDB and Pfam databases, we found that PTBP1 potentially binds to both ENST00000436340 and RAB3B. PTBP1, also known as PTB or heterogeneous nuclear ribonucleoprotein I (hnRNP I), is a ubiquitous RNA-binding protein that regulates alternative splicing [48], mRNA stability [49], and translation initiation [50, 51]. Recently, increasing evidence has reported that PTBP1 can be associated with multiple lncRNAs. For instance, maternally expressed 3 (MEG3) binds to PTBP1 to control Shp mRNA stability and then modulates hepatic BA metabolism [49]. H19 interacts with PTBP1 to reprogram hepatic lipid homeostasis [52]. In the present study, we verified that residues 1008-1671 of PTBP1 intensively interacted with residues 392-868 of ENST00000436340. By treating podocytes with actinomycin D to block the transcriptional process, we observed that ENST00000436340 enhanced PTBP1 binding to RAB3B mRNA to alter RAB3B mRNA stability, revealing that ENST00000436340 may act as a protein scaffold to recruit PTBP1 to RAB3B to promote RAB3B mRNA degradation.

Interestingly, podocytes are insulin sensitive, and evidence has indicated that long-term exposure of podocytes to high glucose induces insulin resistance and leads to impaired function of podocytes [53, 54]. Concomitantly, insulin resistance is an important factor in the development and progression of DKD [55, 56]. In addition, Rab GTPases have been reported to be involved in GLUT4 trafficking [20, 21], which plays an important role in insulin resistance [22]. This finding raises our interest in the role of RAB3B, which regulates GLUT4-mediated insulin resistance in podocytes. By performing a colocalization experiment, we observed colocalization between RAB3B and GLUT4, suggesting that RAB3B might play a role in intracellular GLUT4 trafficking in podocytes. Considering that Rab activity is tightly regulated by cycling between an active GTP-bound and an inactive GDP-bound form [57, 58], we constructed wild-type and dominant-negative mutant (T36N) of RAB3B to further study how RAB3B affects GLUT4 translocation, and we showed that wild-type RAB3B increased insulin-stimulated translocation of GLUT4 to the plasma membrane, while RAB3B T36N blunted the insulin-stimulated translocation of GLUT4 to the plasma membrane, which indicated that the GTPase activity of RAB3B is needed for insulin-regulated GLUT4 translocation, consistent with a previous study [59]. Previous works have shown that chronic exposure of cultured human podocytes to high glucose reduced GLUT4 expression [60]. However, the detailed molecular mechanisms are largely unknown. In the present study, we propose a new opinion that ENST00000436340/RAB3B regulates GLUT4 translocation to participate in podocyte injury in DKD. Recently, single-cell RNA sequencing revealed that RAB3B was downregulated in glomerular disease or podocyte injury models, and knockdown of RAB3B resulted in significant loss and rearrangement of F-actin stress fibers [61], consistent with our results in the present study. Given that actin filaments also participate in the transport of GLUT4 traffic [62, 63], the cytoskeleton rearrangement caused by ENST00000436340-induced RAB3B inhibition may also be responsible for the reduction of GLUT4 translocation to the plasma membrane, revealing that regulation of GLUT4 subcellular localization is a potential therapeutic strategy against insulin resistance in DKD.

In our study, we reported that ENST00000436340 is crucial for human podocyte injury, although this lncRNA primary sequence is expressed specifically in primates. Unlike other noncoding RNAs, such as microRNAs and small nucleolar RNAs, which can exhibit high degrees of conservation across diverse species, most lncRNAs exhibit weak or untraceable primary sequence conservation [64]. Approximately one-third of the lncRNAs are unique to the primate lineage, and only ~12% of human lncRNAs appear to be conserved in other vertebrate species [65]. However, lack of conservation does not mean lack of function [66]. For example, lnc-TSI, a kidney-enriched lncRNA critical for fibrotic kidneys, is poorly conserved in mice [67]. lncRNA UCA1, which is not significantly conserved in mammals, controls human erythroid differentiation [68]. Braveheart, a lncRNA critical for cardiovascular lineage commitment in mice, is not expressed in rats or humans [69]. In addition, certain long functional ncRNAs, such as Xist [66, 70], LINC01018 [71] and LncND [65] are also poorly conserved. Clearly, primary sequence conservation does not invariably predict functional importance.

In conclusion, we identified an ENST00000436340-PTBP1-RAB3B axis in regulating cytoskeleton rearrangement and GLUT4 translocation, leading to podocyte injury (Fig. 8F). The findings of this study have significant implications regarding our understanding of DKD pathogenesis and will provide a basis for the development of more efficient strategies to improve podocyte injury in DKD.

## Supplementary information

Supplementary Fig. 1

Supplementary Fig. 2

Supplementary Fig. 3

Supplementary Fig. 4

Supplementary Fig. 5

Supplementary Fig. 6

Supplementary Fig. legends

Supplementary Table

Original western blots

responses to author rearrangement

## Data Availability

All data generated or analyzed during this study are included either in this article or in the supplementary information files. The RNA sequencing data supporting the findings of this study are openly available in the Gene Expression Omnibus database (GSE199838).
