# Supplementary figures and images for: Long noncoding RNA ENST00000436340 promotes podocyte injury in diabetic kidney disease by facilitating the association of PTBP1 with RAB3B
Source: Cell Death Dis. 2023 Feb 15;14(2):130. doi: 10.1038/s41419-023-05658-7 (PMC9932062; doi:10.1038/s41419-023-05658-7)

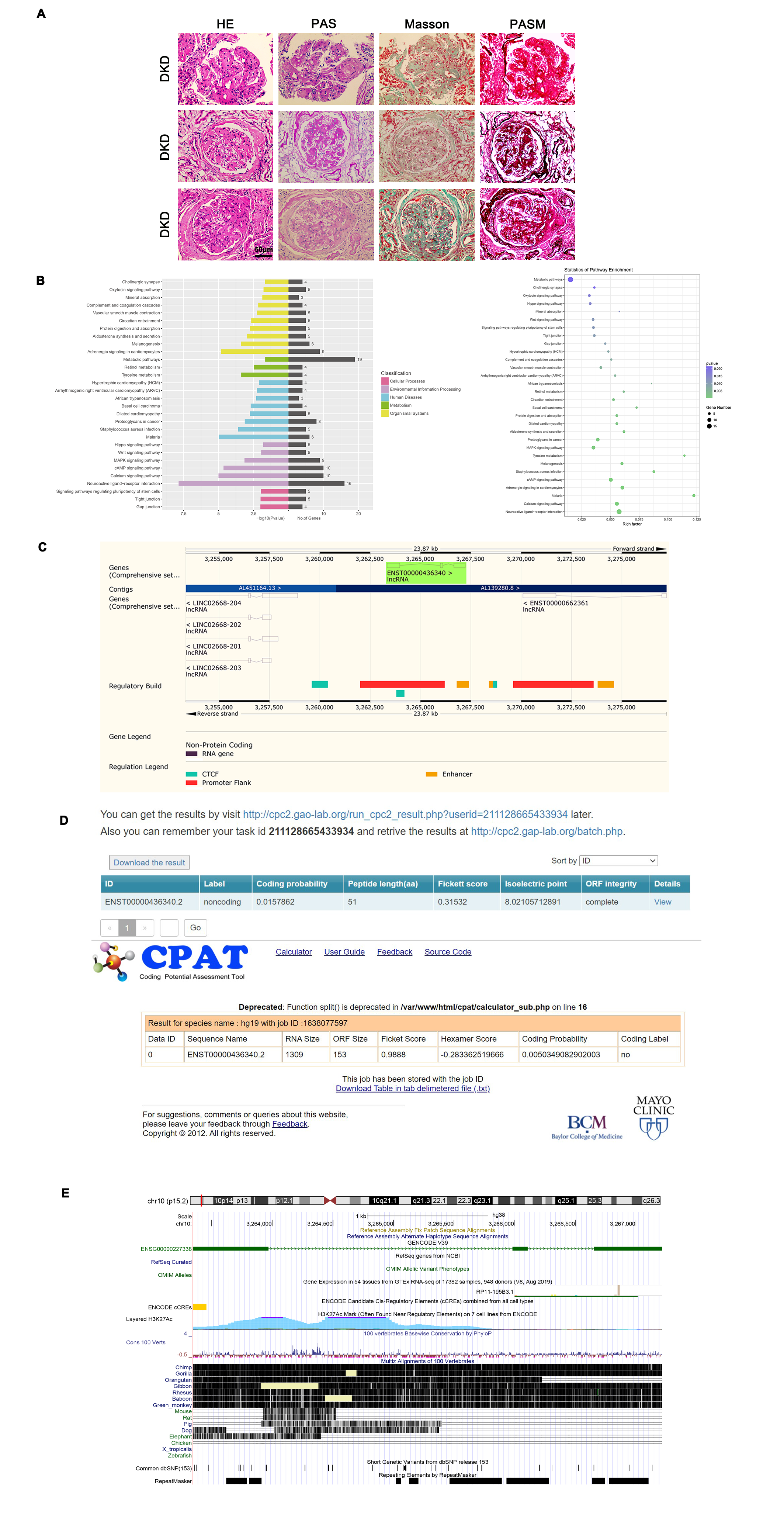

Supplement: Supplementary file 1 — Supplementary Fig. 1 [file 41419_2023_5658_MOESM1_ESM.tif]

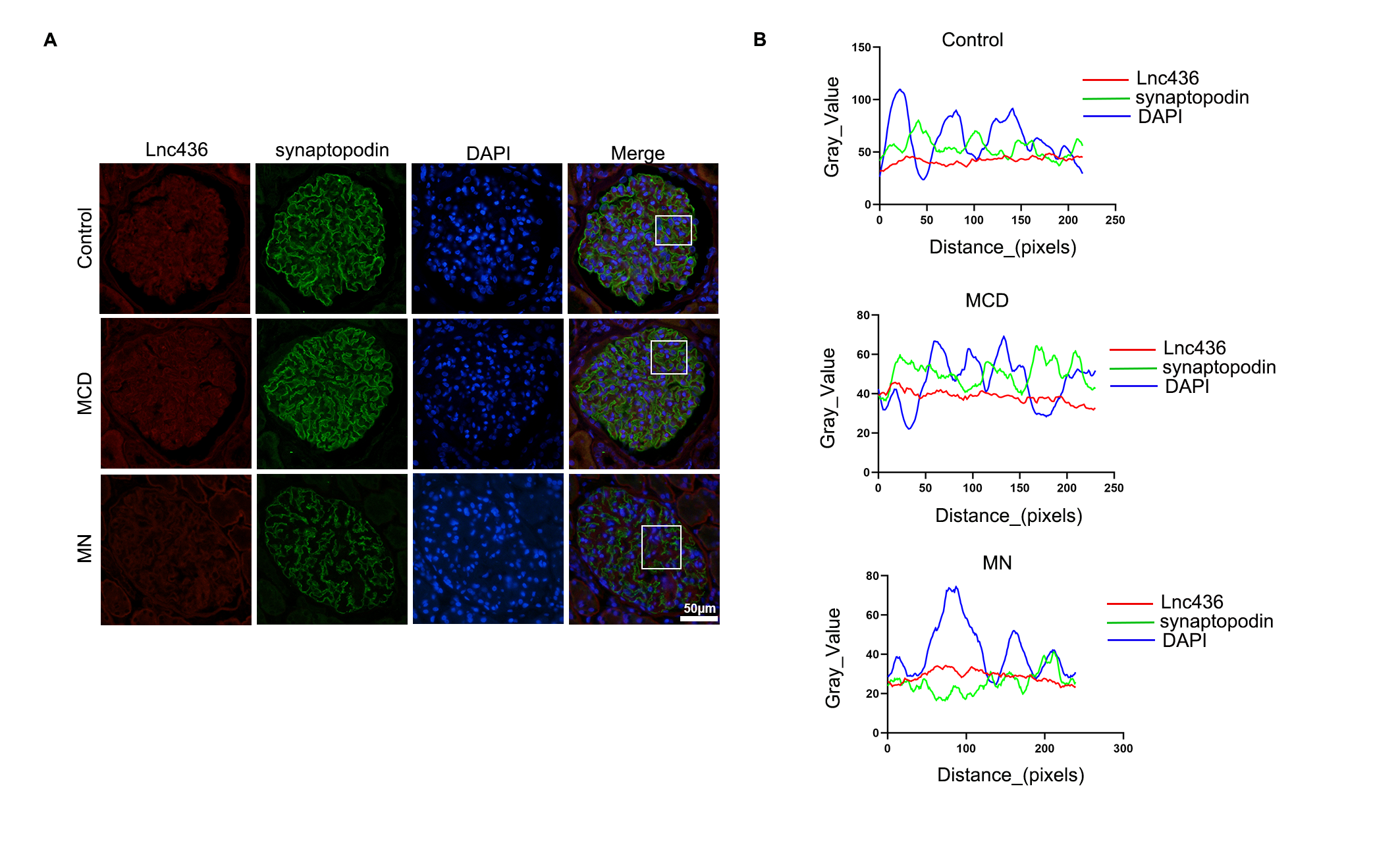

Supplement: Supplementary file 2 — Supplementary Fig. 2 [file 41419_2023_5658_MOESM2_ESM.tif]

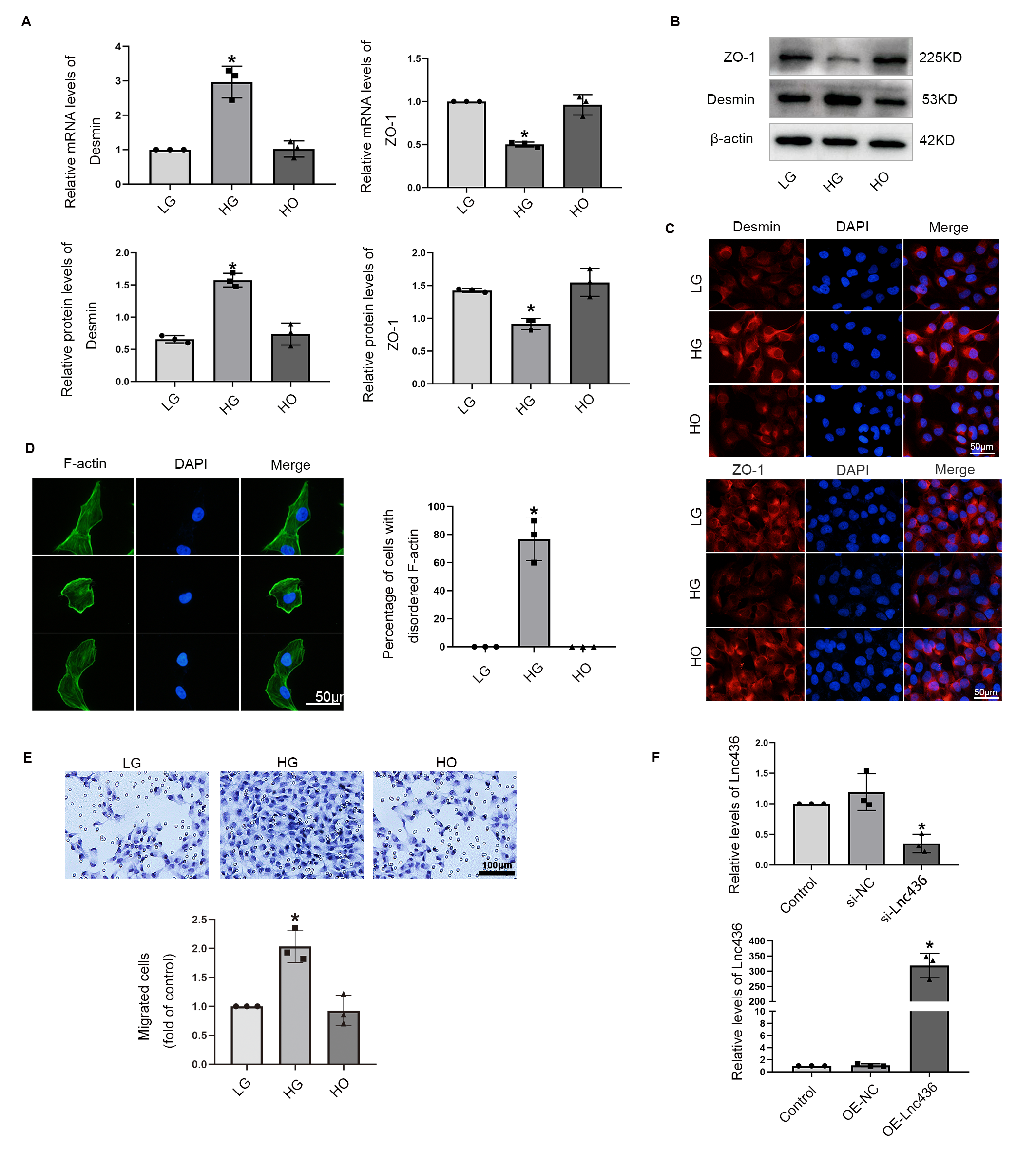

Supplement: Supplementary file 3 — Supplementary Fig. 3 [file 41419_2023_5658_MOESM3_ESM.tif]

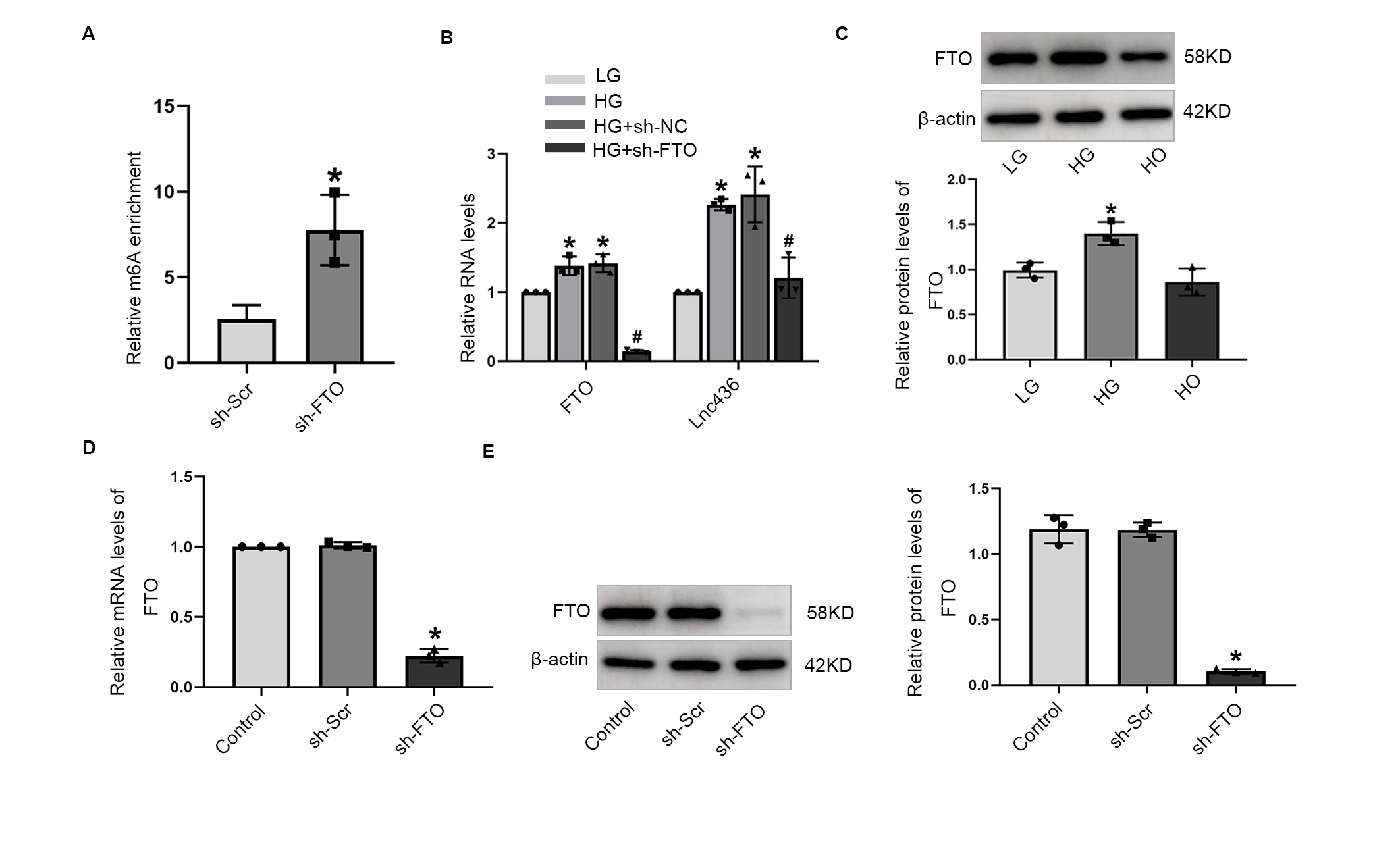

Supplement: Supplementary file 4 — Supplementary Fig. 4 [file 41419_2023_5658_MOESM4_ESM.tif]

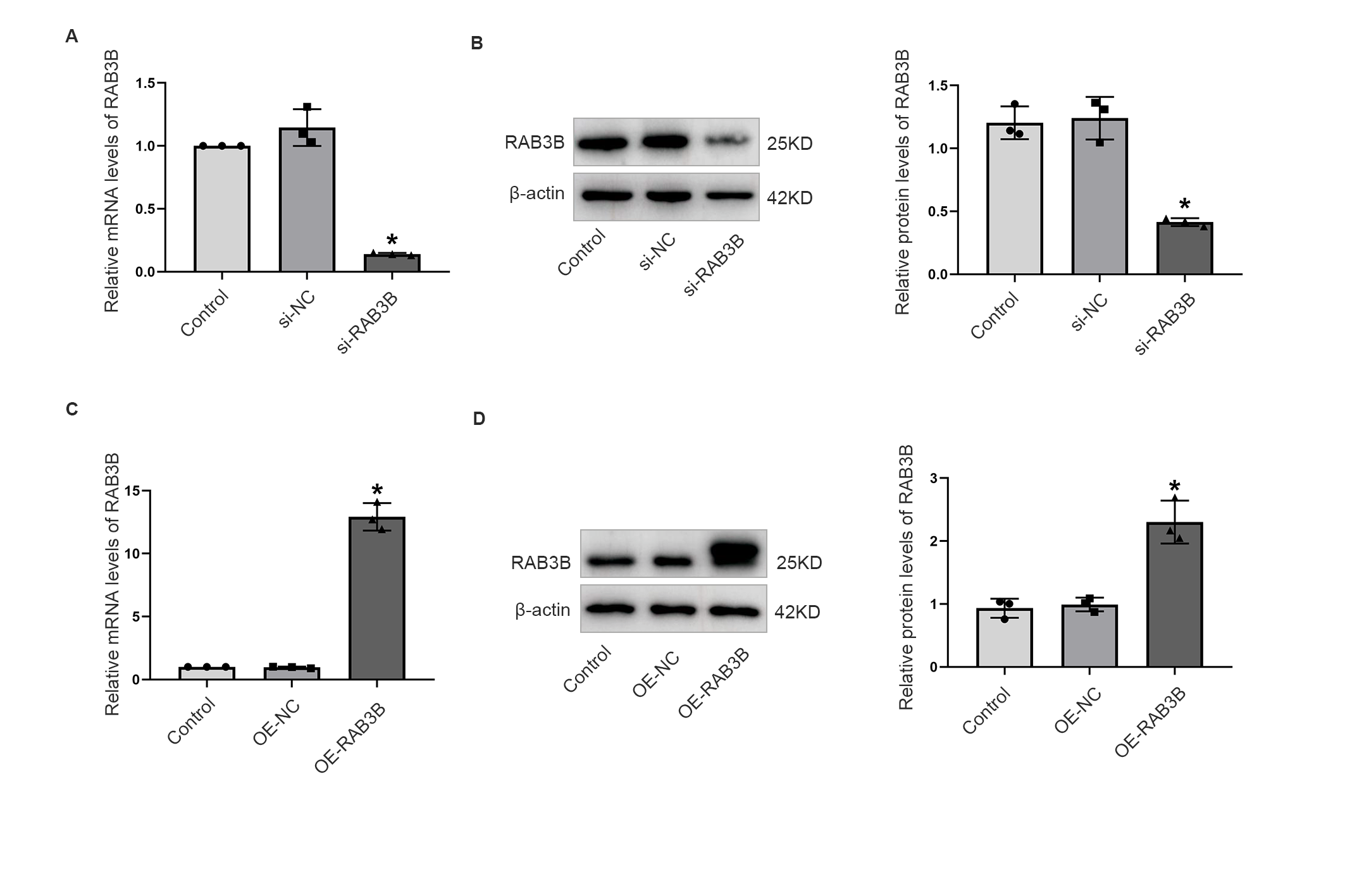

Supplement: Supplementary file 5 — Supplementary Fig. 5 [file 41419_2023_5658_MOESM5_ESM.tif]

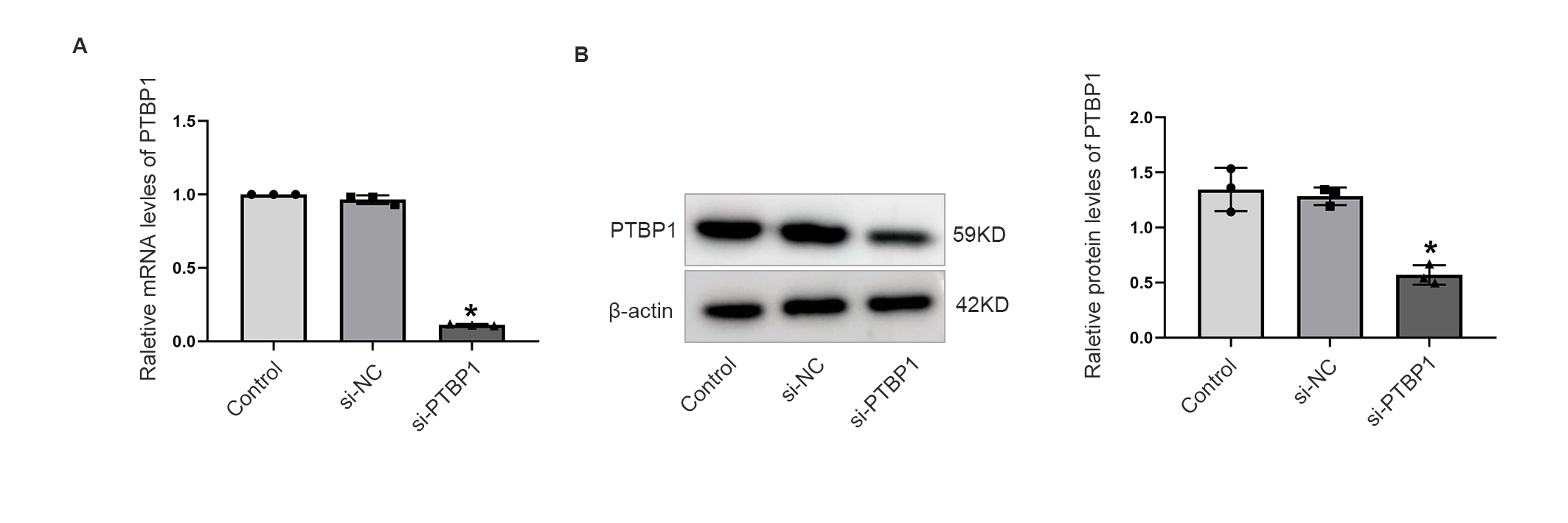

Supplement: Supplementary file 6 — Supplementary Fig. 6 [file 41419_2023_5658_MOESM6_ESM.tif]

Fig. 2E

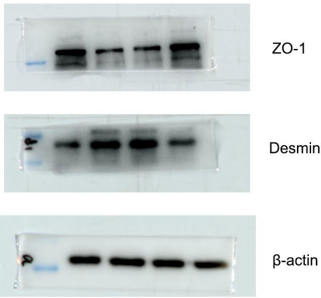

Fig. 2J

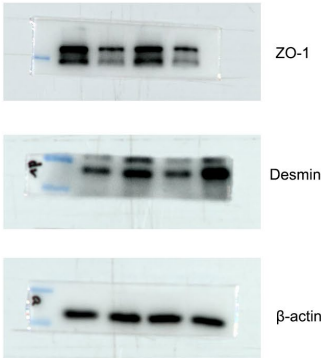

Fig. 3E

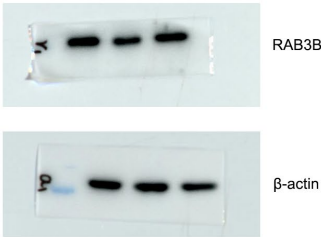

Fig. 3F

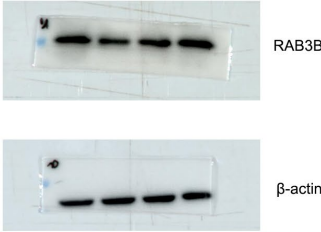

Fig. 3G

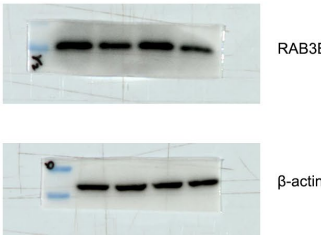

Fig. 4B

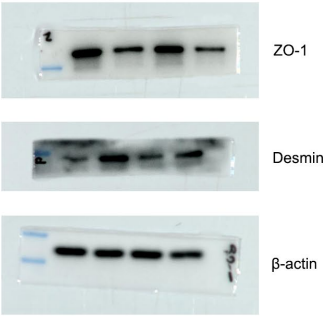

Fig. 4F

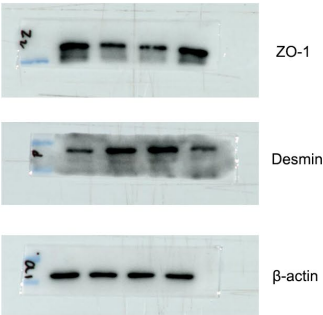

Fig. 5B

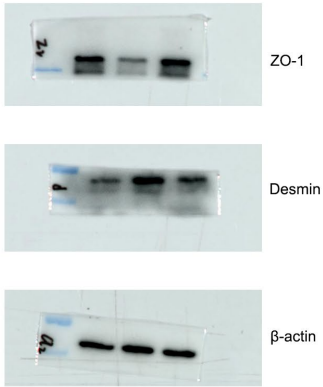

Fig. 6C

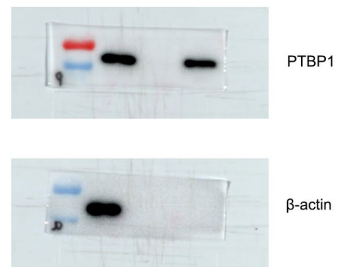

Fig. S3B

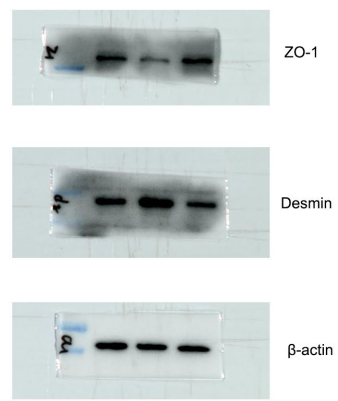

Fig. S4C

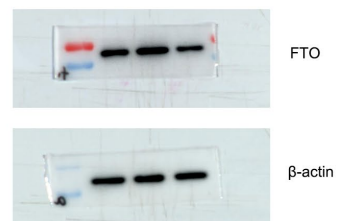

Fig. S4E

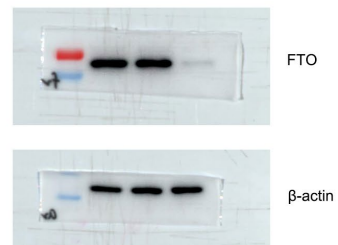

Fig. S5B

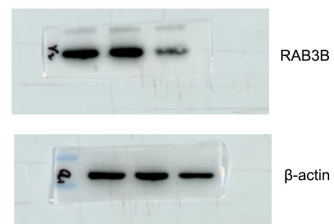

Fig. S5D

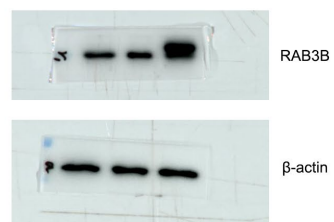

Fig. S6B

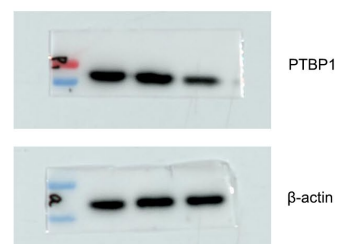

Supplement: Supplementary file 9 — Original western blots [file 41419_2023_5658_MOESM9_ESM.pdf]
