# Supplementary material for: Long noncoding RNA ENST00000436340 promotes podocyte injury in diabetic kidney disease by facilitating the association of PTBP1 with RAB3B
Source: Cell Death Dis. 2023 Feb 15;14(2):130. doi: 10.1038/s41419-023-05658-7 (PMC9932062; doi:10.1038/s41419-023-05658-7)
Supplement: Supplementary file 7 — Supplementary Fig. legends [file 41419_2023_5658_MOESM7_ESM.docx]

**Fig. S1.** (**A**) HE, PAS, and Masson staining indicated the typical pathological changes of kidney in DKD patients used for RNA sequencing. (**B)** KEGG enrichment analyses of differentially expressed genes between normal control and DKD patients. (**C)** Location of ENST00000436340 in human genome. (**D)** The coding potential calculator (CPC2) and coding-potential assessment tool (CPAT) were used to evaluate protein-coding potential of ENST00000436340. (**E)** ENST00000436340 conservation among mammals from UCSC Genome Browser.

**Fig. S2.** (**A**) Representative images of Lnc436 and podocyte-specific marker synaptopodin in the glomeruli of kidney. Arrows indicate colocalization of Lnc436 and synaptopodin. Scale bar=50μm. (**B**) Plots of pixel intensity of white boxed region from merged images (right). MCD: minimal change disease, MN: membranous nephropathy.

**Fig. S3. High glucose induced podocyte injury and cytoskeleton rearrangement**

(**A**) real-time PCR analyses show the mRNA levels of Demin and ZO-1 in different groups of podocytes. (**B**) Western blot analyses show the protein levels of Desmin and ZO-1 in different groups of podocytes. (**C**) Representative immunofluorescence images of Desmin, and ZO-1 in different groups of podocytes. Scale bar = 50μm. (**D**) Representative immunofluorescence images of F-actin in different groups of podocytes. Scale bar = 50μm. (**E**) Representative migration results of podocytes. Scale bar = 100μm. (**F**) The knockdown and overexpression efficiency of Lnc436 in podocytes were examined by real-time PCR. Data are shown as mean ± SD. **P*<0.05 vs LG or Control. The experiment was performed in triplicate. Lnc436: ENST00000436340.

**Fig. S4. FTO is associated with ENST00000436340 upregulation in high glucose-induced podocyte**

(**A**) MeRIP assay was performed to detect the m6A modification of ENST00000436340 in podocytes. *P<0.05 vs sh-NC. (**B**)real-time PCR analyses show the RNA levels of FTO and ENST00000436340 in different groups of podocytes. *P<0.05 vs LG, #P<0.05 vs HG. (**C**) Western blot analyses show the protein levels of FTO in different groups of podocytes. *P<0.05 vs LG. (**D**) Podocytes were treated with FTO shRNA (sh-FTO) or Scramble shRNA (sh-Scr), the knockdown efficiency was examined by real-time PCR. (**E**) Podocytes were treated with FTO shRNA (sh-FTO) or Scramble shRNA (sh-Scr), the knockdown efficiency was examined by Western blot. *P<0.05 vs Control. Data are shown as mean ± SD. The experiment was performed in triplicate.

**Fig. S5.** (**A** and **B**) The podocytes were treated with RAB3B siRNA (si-RAB3B) or negative control (si-NC), the knockdown efficiency was examined by real-time PCR(**A**) and Western blot (**B**). (**C** and **D**) The podocytes were treated with RAB3B overexpression plasmid (OE-RAB3B) or control (OE-NC) vectors, the overexpression efficiency was examined by real-time PCR (**C**) and Western blot (**D**). Data are shown as mean ± SD. **P*<0.05 vs control. The experiment was performed in triplicate.

**Fig. S6**: The podocytes were treated with PTBP1 siRNA (si-PTBP1) or negative control (si-NC), the knockdown efficiency was examined by real-time PCR(**A**) and Western blot (**B**). Data are shown as mean ± SD. **P*<0.05 vs control. The experiment was performed in triplicate.
