# Supplementary material for: Long noncoding RNA ENST00000436340 promotes podocyte injury in diabetic kidney disease by facilitating the association of PTBP1 with RAB3B
Source: Cell Death Dis. 2023 Feb 15;14(2):130. doi: 10.1038/s41419-023-05658-7 (PMC9932062; doi:10.1038/s41419-023-05658-7)
Supplement: Supplementary file 8 — Supplementary Table [file 41419_2023_5658_MOESM8_ESM.docx]

**Table S1**

The target sequences of siRNAs and shRNAs

| Name | Target sequences (5’-3’) |
| --- | --- |
| ENST00000436340 siRNA | CCATCCAAGTCTCTTGCTT |
| RAB3B siRNA | AGUGCAAAGGAGAACAUCAGU |
| PTBP1 siRNA | GCGUCGUCAAAGGAUUCAA |
| GLUT4 siRNA | GCCCUACGUCUUCCUUCUAUU |
| NC siRNA | UUCUCCGAACGUGUCACGU |
| FTO shRNA | GGTGGCAGTGTACAGTTATAG |
| Scramble shRNA | TTCTCCGAACGTGTCACGT |

**Table S2**

The sequences of the primers used in real-time PCR

| Name | Sequence |
| --- | --- |
| ENST00000436340 | F 5’- CCGCACCATCCAAGTCTCTT -3’  R 5’- TCACAAAGTAACCCCTGGATGA -3’ |
| RAB3B | F 5’-ACAACAGCCTATTACCGTGGG -3’  R 5’-CTCAGTGGGAACAACCCTCTC -3’ |
| PTBP1 | F 5’-TACAAAGCGGGGATCTGACG- 3’  R 5’-TCGGCTGTCACCTTTGAACT- 3’ |
| Desmin | F 5’-AGGACCGATTTGCCAGTGAG- 3’  R 5’-CTTGAGGTGCCGGATTTCCT- 3’ |
| ZO-1 | F 5’-CCCCCAACTCAAACCGAAGA- 3’  R 5’-AGATGCTACTTCTGGAGGCTTA- 3’ |
| GLUT4 | F 5’-TGGACGATTCCTCATTGGCG - 3’  R 5’-CAGGTGAGTGGGAGCAATCT - 3’ |
| β-actin | F 5’- CATGTACGTTGCTATCCAGGC- 3’  R 5’-CTCCTTAATGTCACGCACGAT- 3’ |

**Table S3**  Characteristics of Participants

| Group | Gender | Age  (Years) | Scr  (μmol/l) | eGFR  (ml/min/1.73 m^2^) | proteinuria | 24 h  Proteinuria (g) | Pathological  Diagnosis |
| --- | --- | --- | --- | --- | --- | --- | --- |
| DKD | female | 52 | 62 | 100 | +++ | 4.26 | DKD (diffuse and nodular glomerulosclerosis type) |
| DKD | male | 48 | 143 | 50 | +++ | 3.92 | DKD (nodular glomerulosclerosis type) |
| DKD | male | 51 | 146 | 47 | ++ | 3.44 | DKD (nodular glomerulosclerosis type) |
| normal control | male | 35 | 95 | 91 | - | 0.12 | clear cell carcinoma |
| normal control | male | 63 | 133 | 49 | - | 0.08 | clear cell carcinoma |
| normal control | male | 48 | 69.2 | 119 | - | 0.05 | clear cell carcinoma |

**Table S4**

Basal clinical and biochemical characteristics of the Control and DKD patients

| Characteristics | Control  （n=47） | DKD | | P |
| --- | --- | --- | --- | --- |
|  |  | Microalbuminuria（n=25） | Macroalbuminuria（n=23） |  |
| Age, years | 52.96±10.01 | 54.32±6.10 | 53.13±6.36 | 0.723^b^ |
| Men，% | 53.19% | 52.00% | 56.52% | 0.791^a^ |
| SBP，mmHg | 126.43±8.27 | 140.12±10.29* | 146.83±14.03* | <0.001^b^ |
| DBP，mmHg | 77.62±7.59 | 81.60±6.19* | 83.91±6.62* | 0.002^b^ |
| HbAc1，% | 5.21±0.64 | 8.672±1.53* | 8.5±1.35* | <0.001^b^ |
| Serum albumin，g/L | 46.54±1.94 | 40.48±2.41* | 34.28±2.66*^#^ | <0.001^b^ |
| BUN，mmol/L | 4.47±0.78 | 5.93±1.09* | 12.29±4.68*^#^ | <0.001^b^ |
| Serum creatine, μmol/L | 63.57±14.45 | 77.28±12.92* | 133.87±29.18*^#^ | <0.001^b^ |
| Serum uric acid, μmol/L | 278.89±67.19 | 256.88±53.66 | 354.35±114.98*^#^ | 0.002^b^ |
| TC, mmol/L | 4.67±0.81 | 4.79±1.10 | 4.94±0.84 | 0.477^b^ |
| TG，mmol/L | 1.61±0.51 | 2.27±0.55* | 2.53±0.64* | <0.001^b^ |
| LDL-c，mmol/L | 2.95±0.41 | 3.06±0.85 | 3.13±0.30 | 0.129^b^ |
| HDL-c，mmol/L | 1.31±0.23 | 1.14±0.19* | 1.21±0.17* | 0.002^b^ |
| eGFR，mL/min/1.73m^2^ | 108.04±9.64 | 86.44±13.54* | 73.04±17.52*^#^ | <0.001^b^ |
| ACR，mg/g | 9.74±3.41 | 98.72±39.81* | 715.53±271.86*^#^ | <0.001^b^ |

Data with normally distributed are presented as mean ± SD, and data with a skewed distribution are presented as median±interquartile range. *P*<0.05 indicated statistical significance, ^∗^*P*<0.05 *vs* Control, ^#^*P*<0.05 *vs* Microalbuminuria. a represents Chi-square test while b represents one-way analysis of variance (ANOVA).

Abbreviations: SBP: systolic blood pressure, DBP: diastolic blood pressure, HbA1c: glycated hemoglobin, TC: total cholesterol, TG: triglyceride, HDL-c: high-density lipoprotein cholesterol, LDL-c: low-density lipoprotein cholesterol, eGFR: estimated glomerular filtration rate; ACR: urine albumin/creatinine.

**Table S5**

Correlation analysis between serum ENST00000436340 levels and other variables in patients with DKD

| Characteristics | Spearman correlation（r_s_） | P |
| --- | --- | --- |
| Age, years | -0.188 | 0.201 |
| Men，% | 0.235 | 0.108 |
| SBP，mmHg | 0.099 | 0.503 |
| DBP，mmHg | 0.206 | 0.161 |
| HbAc1，% | 0.007 | 0.963 |
| Serum albumin，g/L | -0.424** | 0.003 |
| BUN，mmol/L | 0.397** | 0.005 |
| Serum creatine, μmol/L | 0.424** | 0.003 |
| Serum uric acid, μmol/L | 0.234 | 0.109 |
| TC, mmol/L | 0.065 | 0.662 |
| TG，mmol/L | 0.038 | 0.798 |
| LDL-c，mmol/L | 0.034 | 0.817 |
| HDL-c，mmol/L | 0.081 | 0.586 |
| eGFR，mL/min/1.73m^2^ | -0.156 | 0.291 |
| ACR，mg/g | 0.546*** | <0.001 |

****P*<0.001，***P*<0.01

**Table S6**

LncRNA-mRNAs for cis-regulation and trans-regulation.

Cis-relation

| lncRNAs | Target mRNAs |
| --- | --- |
| NR_135824.1 | MST1L |
| NR_003578.1 | ZNF888 |
| ENST00000458139.1 | LMOD1 |
| ENST00000418393.1 | KRT10 |
| ENST00000533082.1 | LOC102724957 |

Trans-relation

| lncRNAs | Target mRNAs |
| --- | --- |
| ENST00000379317.1 | CRX、TRIM58、PRELP、PTGIS、ALPP、PRDM14、FGL2、RAB3B |
| ENST00000414159.2 | CRX、PTGIS、TNS4 |
| ENST00000436340.1 | RAB3B |
| ENST00000451362.1 | CRX、RAB3B |
| ENST00000458139.1 | LMOD1 |
| ENST00000532153.1 | CRX、TRIM58、PRELP、PTGIS、ZFR2、PRDM14、RAB3B |
| ENST00000563495.1 | CRX、TRIM58、PRELP、PRDM14、FGL2、RAB3B、TNS4 |
| NR_003570.2 | CRX、PRR23C、PTGIS、TNS4 |
| NR_003578.1 | PTGIS |
| NR_040021.1 | CRX、TRIM58、PRELP、PTGIS、RAB3B、 |
| NR_120649.1 | FGL2 |
| NR_135293.1 | CRX、TRIM58、PRELP、FGL2、RAB3B |
