# Supplementary material for: Long noncoding RNA ENST00000436340 promotes podocyte injury in diabetic kidney disease by facilitating the association of PTBP1 with RAB3B
Source: Cell Death Dis. 2023 Feb 15;14(2):130. doi: 10.1038/s41419-023-05658-7 (PMC9932062; doi:10.1038/s41419-023-05658-7)
Supplement: Supplementary file 10 — responses to author rearrangement [file 41419_2023_5658_MOESM10_ESM.pdf]

Re: Changes to authorship

"蒙娜丽莎" <1847116099@qq.com>

收件人: "Zhimei Lv" <sdlvzhimei@163.com>

时　间: 2023-2-1 23:03:17

附　件:

I agree.

---Original---

From: "Zhimei Lv"<sdlvzhimei@163.com>
Date: Wed, Feb 1, 2023 23:01 PM
To: "351214972"<351214972@qq.com>;"1847116099"<1847116099@qq.com>;"fanxiaoting0903"<fanxiaoting0903@163.com>;"zjh57renal"<zjh57renal@126.com>;"wang22936"<wang22936@163.com>;"CHENchm"<CHENchm@126.com>;"17865585878"<17865585878@163.com>;"zp1536032406"<zp1536032406@163.com>;"zhangtw3112021"<zhangtw3112021@163.com>;"sd_wangrong"<sd_wangrong@163.com>;"htt2112021"<htt2112021@126.com>;"893087903"<893087903@qq.com>;
Subject: Changes to authorship

Changes to authorship

I am writing to request and agree to a change in the authorship of our article entitled “Long noncoding RNA ENST00000436340 promotes podocyte injury in diabetic kidney disease by facilitating the association of PTBP1 with RAB3B”, as described below.

The original authorship was:

Jinxiu Hu , Yue Liu , Qimeng Wang , Junhui Zhen , Cheng Wang , Huimin Chen , Yingxiao Liu , Ping Zhou , Tingwei Zhang , Tongtong Huang , Rong Wang, and Zhimei Lv

The new author list will be:

Jinxiu Hu, Qimeng Wang, Xiaoting Fan, Junhui Zhen, Cheng Wang, Huimin Chen, Yingxiao Liu, Ping Zhou, Tingwei Zhang, Tongtong Huang, Rong Wang, and Zhimei Lv

Please reply to this email confirming that you agreed with the rearrangement of the names.

Kind regards,

Zhimei Lv

Changes to authorship

"错觉" <351214972@qq.com>

收件人："Zhimei Lv" <sdlvzhimei@163.com>

时　间：2023-2-1 23:11:07

附　件：

I agree

---原始邮件---

发件人：Zhimei Lv"

发送时间："undefined"

收件人："351214972"<351214972@qq.com>,"1847116099" <1847116099@qq.com>,"fanxiaoting0903","zjh57renal","wang22936","CHENchm","17865585878" <17865585878@163.com>,"zp1536032406","zhangtw3112021","sd_wangrong","htt2112021","893087903" <893087903@qq.com>

主题：Changes to authorship

Changes to authorship

I am writing to request and agree to a change in the authorship of our article entitled “Long noncoding RNA ENST00000436340 promotes podocyte injury in diabetic kidney disease by facilitating the association of PTBP1 with RAB3B”, as described below.

The original authorship was:

Jinxiu Hu , Yue Liu , Qimeng Wang , Junhui Zhen , Cheng Wang , Huimin Chen , Yingxiao Liu , Ping Zhou , Tingwei Zhang , Tongtong Huang , Rong Wang, and Zhimei Lv

The new author list will be:

Jinxiu Hu, Qimeng Wang, Xiaoting Fan, Junhui Zhen, Cheng Wang, Huimin Chen, Yingxiao Liu, Ping Zhou, Tingwei Zhang, Tongtong Huang, Rong Wang, and Zhimei Lv

Please reply to this email confirming that you agreed with the rearrangement of the names.

Kind regards,

Zhimei Lv

Re: Changes to authorship

"范晓亭" <fanxiaoting0903@163.com>

收件人: "Zhimei Lv" <sdlvzhimei@163.com>

时　间: 2023-2-2 9:05:42

附　件:

Dear professor Lv:

I've received this email and I agree with the changes to authorship.I really appreciate that you gave me the chance to participate in this article.

Sweet dreams!

Students Xiaoting Fan

---- Replied Message ----

From	Zhimei Lv<sdlvzhimei@163.com>
Date	02/01/2023 23:01
To	351214972<351214972@qq.com>, 1847116099<1847116099@qq.com>, fanxiaoting0903<fanxiaoting0903@163.com>, zjh57renal<zjh57renal@126.com>, wang22936<wang22936@163.com>, CHENchm<chenchm@126.com>, 17865585878<17865585878@163.com>, zp1536032406<zp1536032406@163.com>, zhangtw3112021<zhangtw3112021@163.com>, sd_wangrong<sd_wangrong@163.com>, htt2112021<htt2112021@126.com>, 893087903<893087903@qq.com>
Subject	Changes to authorship

Changes to authorship

I am writing to request and agree to a change in the authorship of our article entitled "Long noncoding RNA ENST00000436340 promotes podocyte injury in diabetic kidney disease by facilitating the association of PTBP1 with RAB3B", as described below.

The original authorship was:

Jinxiu Hu , Yue Liu , Qimeng Wang , Junhui Zhen , Cheng Wang , Huimin Chen , Yingxiao Liu , Ping Zhou , Tingwei Zhang , Tongtong Huang , Rong Wang, and Zhimei Lv

The new author list will be:

Jinxiu Hu, Qimeng Wang, Xiaoting Fan, Junhui Zhen, Cheng Wang, Huimin Chen, Yingxiao Liu, Ping Zhou, Tingwei Zhang, Tongtong Huang, Rong Wang, and Zhimei Lv

Please reply to this email confirming that you agreed with the rearrangement of the names.

Kind regards,
Zhimei Lv

Re: Changes to authorship

"zjh57renal@126.com" <zjh57renal@126.com>

收件人：sdlvzhimei <sdlvzhimei@163.com>

时　间：2023-2-1 23:24:21

附　件：

Dear Professor LV,

I agree with the changes to authorship of the article entitled "Long noncoding RNA ENST00000436340 promotes podocyte injury in diabetic kidney disease by facilitating the association of PTBP1 with RAB3B".

With best regards
Junhui Zhen

zjh57renal@126.com

邮箱：zjh57renal@126.com

---- Replied Message ----

From	Zhimei Lv<sdlvzhimei@163.com>
Date	02/01/2023 23:01
To	351214972@qq.com<351214972@qq.com>、1847116099@qq.com<1847116099@qq.com>、fanxiaoting0903@163.com<fanxiaoting0903@163.com>、zjh57renal@126.com<zjh57renal@126.com>、wang22936@163.com<wang22936@163.com>、CHENchm@126.com<CHENchm@126.com>、17865585878@163.com<17865585878@163.com>、zp1536032406@163.com<zp1536032406@163.com>、zhangtw3112021@163.com<zhangtw3112021@163.com>、sd_wangrong@163.com<sd_wangrong@163.com>、htt2112021@126.com<htt2112021@126.com>、893087903@qq.com<893087903@qq.com>
Subject	Changes to authorship

Changes to authorship

I am writing to request and agree to a change in the authorship of our article entitled “Long noncoding RNA ENST00000436340 promotes podocyte injury in diabetic kidney disease by facilitating the association of PTBP1 with RAB3B”, as described below.

The original authorship was:

Jinxiu Hu , Yue Liu , Qimeng Wang , Junhui Zhen , Cheng Wang , Huimin Chen , Yingxiao Liu , Ping Zhou , Tingwei Zhang , Tongtong Huang , Rong Wang, and Zhimei Lv

The new author list will be:

Jinxiu Hu, Qimeng Wang, Xiaoting Fan, Junhui Zhen, Cheng Wang, Huimin Chen, Yingxiao Liu, Ping Zhou, Tingwei Zhang, Tongtong Huang, Rong Wang, and Zhimei Lv

Please reply to this email confirming that you agreed with the rearrangement of the names.

Kind regards,
Zhimei Lv

Re:Changes to authorship

"王呈" <wang22936@163.com>
收件人: "Zhimei Lv" <sdlvzhimei@163.com>
时　间: 2023-2-2 14:14:17
附　件:

I AGREE

At 2023-02-01 23:01:10, "Zhimei Lv" <sdlvzhimei@163.com> wrote:

> Changes to authorship
>
> I am writing to request and agree to a change in the authorship of our article entitled "Long noncoding RNA ENST00000436340 promotes podocyte injury in diabetic kidney disease by facilitating the association of PTBP1 with RAB3B", as described below.
>
> The original authorship was:
>
> Jinxiu Hu , Yue Liu , Qimeng Wang , Junhui Zhen , Cheng Wang , Huimin Chen , Yingxiao Liu , Ping Zhou , Tingwei Zhang , Tongtong Huang , Rong Wang, and Zhimei Lv
>
> The new author list will be:
>
> Jinxiu Hu, Qimeng Wang, Xiaoting Fan, Junhui Zhen, Cheng Wang, Huimin Chen, Yingxiao Liu, Ping Zhou, Tingwei Zhang, Tongtong Huang, Rong Wang, and Zhimei Lv
>
> Please reply to this email confirming that you agreed with the rearrangement of the names.
>
> Kind regards,
> Zhimei Lv

Re: Changes to authorship

chenchm <chenchm@126.com>

收件人: "sdlvzhimei@163.com" <sdlvzhimei@163.com>

时　间: 2023-2-2 14:14:32

附　件:

I agree with the changes to authorship of the article.

chenchm

chenchm@126.com

---- Replied Message ----

From	Zhimei Lv<sdlvzhimei@163.com>
Date	2/1/2023 23:01
To	<351214972@qq.com> ,<br><1847116099@qq.com> ,<br><fanxiaoting0903@163.com> ,<br><zjh57renal@126.com> ,<br><wang22936@163.com> ,<br><CHENchm@126.com> ,<br><17865585878@163.com> ,<br><zp1536032406@163.com> ,<br><zhangtw3112021@163.com> ,<br><sd_wangrong@163.com> ,<br><htt2112021@126.com> ,<br><893087903@qq.com>
Subject	Changes to authorship

Changes to authorship

I am writing to request and agree to a change in the authorship of our article entitled “Long noncoding RNA ENST00000436340 promotes podocyte injury in diabetic kidney disease by facilitating the association of PTBP1 with RAB3B”, as described below.

The original authorship was:

Jinxiu Hu , Yue Liu , Qimeng Wang , Junhui Zhen , Cheng Wang , Huimin Chen , Yingxiao Liu , Ping Zhou , Tingwei Zhang , Tongtong Huang , Rong Wang, and Zhimei Lv

The new author list will be:

Jinxiu Hu, Qimeng Wang, Xiaoting Fan, Junhui Zhen, Cheng Wang, Huimin Chen, Yingxiao Liu, Ping Zhou, Tingwei Zhang, Tongtong Huang, Rong Wang, and Zhimei Lv

Please reply to this email confirming that you agreed with the rearrangement of the names.

Kind regards,
Zhimei Lv

Re: Changes to authorship

17865585878 <17865585878@163.com>

收件人: "Zhimei Lv" <sdlvzhimei@163.com>

时　间: 2023-2-2 10:28:02

附　件:

Notified and agree，thanks a lot.

---- Replied Message ----

From	Zhimei Lv<sdlvzhimei@163.com>
Date	02/01/2023 23:01
To	351214972<351214972@qq.com>, 1847116099<1847116099@qq.com>, fanxiaoting0903<fanxiaoting0903@163.com>, zjh57renal<zjh57renal@126.com>, wang22936<wang22936@163.com>, CHENchm<chenchm@126.com>, 17865585878<17865585878@163.com>, zp1536032406<zp1536032406@163.com>, zhangtw3112021<zhangtw3112021@163.com>, sd_wangrong<sd_wangrong@163.com>, htt2112021<htt2112021@126.com>, 893087903<893087903@qq.com>
Subject	Changes to authorship

Changes to authorship

I am writing to request and agree to a change in the authorship of our article entitled “Long noncoding RNA ENST00000436340 promotes podocyte injury in diabetic kidney disease by facilitating the association of PTBP1 with RAB3B”, as described below.

The original authorship was:

Jinxiu Hu , Yue Liu , Qimeng Wang , Junhui Zhen , Cheng Wang , Huimin Chen , Yingxiao Liu , Ping Zhou , Tingwei Zhang , Tongtong Huang , Rong Wang, and Zhimei Lv

The new author list will be:

Jinxiu Hu, Qimeng Wang, Xiaoting Fan, Junhui Zhen, Cheng Wang, Huimin Chen, Yingxiao Liu, Ping Zhou, Tingwei Zhang, Tongtong Huang, Rong Wang, and Zhimei Lv

Please reply to this email confirming that you agreed with the rearrangement of the names.

Kind regards,

Zhimei Lv

Re: Changes to authorship

"学生周萍" <zp1536032406@163.com>
收件人：sdlvzhimei <sdlvzhimei@163.com>
时　间：2023-2-2 9:32:45
附　件：

Dear Professor LV,

I agree with the changes to authorship of the article entitled "Long noncoding RNA ENST00000436340 promotes podocyte injury in diabetic kidney disease by facilitating the association of PTBP1 with RAB3B".

With best regards
+Ping Zhou

---- Replied Message ----

From	Zhimei Lv<sdlvzhimei@163.com>
Date	02/01/2023 23:01
To	351214972@qq.com、1847116099@qq.com、fanxiaoting0903@163.com、zjh57renal@126.com、wang22936@163.com、CHENchm@126.com、17865585878@163.com、zp1536032406@163.com、zhangtw3112021@163.com、sd_wangrong@163.com、htt2112021@126.com、893087903@qq.com
Subject	Changes to authorship

Changes to authorship

I am writing to request and agree to a change in the authorship of our article entitled “Long noncoding RNA ENST00000436340 promotes podocyte injury in diabetic kidney disease by facilitating the association of PTBP1 with RAB3B”, as described below.

The original authorship was:

Jinxiu Hu , Yue Liu , Qimeng Wang , Junhui Zhen , Cheng Wang , Huimin Chen , Yingxiao Liu , Ping Zhou , Tingwei Zhang , Tongtong Huang , Rong Wang, and Zhimei Lv

The new author list will be:

Jinxiu Hu, Qimeng Wang, Xiaoting Fan, Junhui Zhen, Cheng Wang, Huimin Chen, Yingxiao Liu, Ping Zhou, Tingwei Zhang, Tongtong Huang, Rong Wang, and Zhimei Lv

Please reply to this email confirming that you agreed with the rearrangement of the names.

Kind regards,

Zhimei Lv

Re: Changes to authorship

"zhangtw3112021@163.com" <zhangtw3112021@163.com>

收件人：sdlvzhimei <sdlvzhimei@163.com>

时　间：2023-2-1 23:23:40

附　件：

Dear Professor LV,

I agree with the changes to authorship of the article entitled "Long noncoding RNA ENST00000436340 promotes podocyte injury in diabetic kidney disease by facilitating the association of PTBP1 with RAB3B".

With best regards
Tingwei Zhang

zhangtw3112021@163.com

邮箱：zhangtw3112021@163.com

---- Replied Message ----

From	Zhimei Lv<sdlvzhimei@163.com>
Date	02/01/2023 23:01
To	351214972@qq.com<351214972@qq.com>、1847116099@qq.com<1847116099@qq.com>、fanxiaoting0903@163.com<fanxiaoting0903@163.com>、zjh57renal@126.com<zjh57renal@126.com>、wang22936@163.com<wang22936@163.com>、CHENchm@126.com<CHENchm@126.com>、17865585878@163.com<17865585878@163.com>、zp1536032406@163.com<zp1536032406@163.com>、zhangtw3112021@163.com<zhangtw3112021@163.com>、sd_wangrong@163.com<sd_wangrong@163.com>、htt2112021@126.com<htt2112021@126.com>、893087903@qq.com<893087903@qq.com>
Subject	Changes to authorship

Changes to authorship

I am writing to request and agree to a change in the authorship of our article entitled “Long noncoding RNA ENST00000436340 promotes podocyte injury in diabetic kidney disease by facilitating the association of PTBP1 with RAB3B”, as described below.

The original authorship was:

Jinxiu Hu , Yue Liu , Qimeng Wang , Junhui Zhen , Cheng Wang , Huimin Chen , Yingxiao Liu , Ping Zhou , Tingwei Zhang , Tongtong Huang , Rong Wang, and Zhimei Lv

The new author list will be:

Jinxiu Hu, Qimeng Wang, Xiaoting Fan, Junhui Zhen, Cheng Wang, Huimin Chen, Yingxiao Liu, Ping Zhou, Tingwei Zhang, Tongtong Huang, Rong Wang, and Zhimei Lv

Please reply to this email confirming that you agreed with the rearrangement of the names.

Kind regards,
Zhimei Lv

Re: Changes to authorship

"htt2112021@126.com" <htt2112021@126.com>

收件人：sdlvzhimei <sdlvzhimei@163.com>

时　间：2023-2-1 23:22:58

附　件：

Dear Professor LV,

I agree with the changes to authorship of the article entitled "Long noncoding RNA ENST00000436340 promotes podocyte injury in diabetic kidney disease by facilitating the association of PTBP1 with RAB3B".

With best regards
+Tongtong Huang

htt2112021@126.com

邮箱：htt2112021@126.com

---- Replied Message ----

From	Zhimei Lv<sdlvzhimei@163.com>
Date	02/01/2023 23:01
To	351214972@qq.com<351214972@qq.com>、1847116099@qq.com<1847116099@qq.com>、fanxiaoting0903@163.com<fanxiaoting0903@163.com>、zjh57renal@126.com<zjh57renal@126.com>、wang22936@163.com<wang22936@163.com>、CHENchm@126.com<CHENchm@126.com>、17865585878@163.com<17865585878@163.com>、zp1536032406@163.com<zp1536032406@163.com>、zhangtw3112021@163.com<zhangtw3112021@163.com>、sd_wangrong@163.com<sd_wangrong@163.com>、htt2112021@126.com<htt2112021@126.com>、893087903@qq.com<893087903@qq.com>
Subject	Changes to authorship

Changes to authorship

I am writing to request and agree to a change in the authorship of our article entitled “Long noncoding RNA ENST00000436340 promotes podocyte injury in diabetic kidney disease by facilitating the association of PTBP1 with RAB3B”, as described below.

The original authorship was:

Jinxiu Hu , Yue Liu , Qimeng Wang , Junhui Zhen , Cheng Wang , Huimin Chen , Yingxiao Liu , Ping Zhou , Tingwei Zhang , Tongtong Huang , Rong Wang, and Zhimei Lv

The new author list will be:

Jinxiu Hu, Qimeng Wang, Xiaoting Fan, Junhui Zhen, Cheng Wang, Huimin Chen, Yingxiao Liu, Ping Zhou, Tingwei Zhang, Tongtong Huang, Rong Wang, and Zhimei Lv

Please reply to this email confirming that you agreed with the rearrangement of the names.

Kind regards,
Zhimei Lv

Re: Changes to authorship

"sd_wangrong@163.com" <sd_wangrong@163.com>

收件人：sdlvzhimei <sdlvzhimei@163.com>

时　间：2023-2-1 23:25:50

附　件:

I agree with the changes to authorship.

sd_wangrong@163.com

邮箱：sd_wangrong@163.com

---- Replied Message ----

From	Zhimei Lv<sdlvzhimei@163.com>
Date	02/01/2023 23:01
To	351214972@qq.com<351214972@qq.com>、1847116099@qq.com<1847116099@qq.com>、fanxiaoting0903@163.com<fanxiaoting0903@163.com>、zjh57renal@126.com<zjh57renal@126.com>、wang22936@163.com<wang22936@163.com>、CHENchm@126.com<CHENchm@126.com>、17865585878@163.com<17865585878@163.com>、zp1536032406@163.com<zp1536032406@163.com>、zhangtw3112021@163.com<zhangtw3112021@163.com>、sd_wangrong@163.com<sd_wangrong@163.com>、htt2112021@126.com<htt2112021@126.com>、893087903@qq.com<893087903@qq.com>
Subject	Changes to authorship

Changes to authorship

I am writing to request and agree to a change in the authorship of our article entitled “Long noncoding RNA ENST00000436340 promotes podocyte injury in diabetic kidney disease by facilitating the association of PTBP1 with RAB3B”, as described below.

The original authorship was:

Jinxiu Hu , Yue Liu , Qimeng Wang , Junhui Zhen , Cheng Wang , Huimin Chen , Yingxiao Liu , Ping Zhou , Tingwei Zhang , Tongtong Huang , Rong Wang, and Zhimei Lv

The new author list will be:

Jinxiu Hu, Qimeng Wang, Xiaoting Fan, Junhui Zhen, Cheng Wang, Huimin Chen, Yingxiao Liu, Ping Zhou, Tingwei Zhang, Tongtong Huang, Rong Wang, and Zhimei Lv

Please reply to this email confirming that you agreed with the rearrangement of the names.

Kind regards,
Zhimei Lv
